# Novel Diketopyrrolopyrrole-Based π-Conjugated Molecules Synthesized Via One-Pot Direct Arylation Reaction

**DOI:** 10.3390/molecules24091760

**Published:** 2019-05-07

**Authors:** Hui Liu, Xiao-Feng Zhang, Jing-Zhao Cheng, Ai-Guo Zhong, He-Rui Wen, Shi-Yong Liu

**Affiliations:** 1 School of Metallurgical and Chemical Engineering, Jiangxi University of Science and Technology, Ganzhou 341000, China; liuhui666753@163.com (H.L.); 13398445081@163.com (X.-F.Z.); cjz19960724@163.com (J.-Z.C.); 2 Department of Chemistry, Taizhou University, Taizhou 317000, China; xg2268@163.com

**Keywords:** diketopyrrolopyrrole (DPP), direct arylation, π-conjugated molecules, cyclic voltammetry, atom economy

## Abstract

Diketopyrrolopyrrole (DPP) is an important type of π-conjugated building block for high-performance organic electronic materials. DPP-based conjugated materials are usually synthesized via Suzuki, Stille, or Negishi cross-coupling reactions, which require organometallic precursors. In this paper, a series of novel phenyl-cored DPP molecules, including five *meta*-phenyl-cored molecules and four *para*-phenyl-cored molecules, have been synthesized in moderate to good yields, in a facile manner, through the Pd-catalyzed direct arylation of C–H bonds, and their optoelectrical properties have been investigated in detail. All new molecules have been fully characterized by NMR, MALDI-TOF MS, elemental analysis, UV–visible spectroscopy, and cyclic voltammetry. This synthetic strategy has evident advantages of atom- and step-economy and low cost, compared with traditional cross-coupling reactions.

## 1. Introduction

π-Conjugated materials play an irreplaceable role in the field of optics and organic electronics. π-Conjugated organic materials can be divided into polymers (molecular weight MW > 10,000), oligomers (MW > 1000) and small molecules (MW < 1000) [1]. Since the discovery of polyacetylene in 1976 [2], π-conjugated polymers, oligomers, and small molecules have drawn extensive attention from researchers. Essentially, the polymers are mixtures of various polymeric chains with different MWs and polydispersity index (PDI) greater than 1. Although the small molecules have defined structures, their conjugation lengths and molecular weights are limited. Nevertheless, the oligomers can be regarded as a kind of special polymer with monodispersity (PDI = 1) and defined structure which might combine the advantages of polymers and small molecules while overcoming their individual shortcomings [3,4]. Therefore, oligomers with defined structure and high molecular weight are expected to be ideal choices for organic π-conjugated functional materials.

Since the discovery of diketopyrrolopyrroles (DPPs) by Farnum [5] et al. in 1974, a large number of DPP-based conjugated molecules or polymers have been synthesized by researchers. Thienyl-flanked DPP (TDPP) [6] has become a popular building block, with a planar structure and being amenable to easy structural modifications [7]. The electron-rich thiophene can give rise to strong intramolecular charge transfer due to the electron-deficient DPP core and intermolecular π–π stacking, which improves the optical and electrochemical properties. Small molecules based on DPP [8,9,10,11], core-modified with two thiophene rings, have also been explored in Organic Photovoltaic Cells (OPVs). Conjugated molecules based on TDPP can be simultaneously used as electron donors and acceptors with bipolar charge-transporting properties. The α-C–H bonds on thiophene rings can be directly arylated by C–X (X = Br or I) under palladium catalysis [12,13,14,15,16], due to the ease of palladation through a concerted metalation–deprotonation (CMD) pathway [17], leading to activation of the C–H bonds on 2/5 positions on the thiophene rings.

DPP-based conjugated materials are usually synthesized by traditional cross-coupling reactions, such as Kumada [18], Suzuki [19], Stille [20,21], and Yamamoto [22], which usually involve multiple steps and pre-functionalization of substrates with C–M bonds, a stoichiometric amount of Ni(0) catalyst, and low overall yields. Direct C–H arylation, as an atom- and step-economic synthetic strategy [23,24,25,26,27,28,29,30,31,32], will overcome these shortcomings and avoid the tedious synthetic steps with higher atomic economy and easier purification compared with the traditional C–C couplings [33].

In continuation of our interest in the atom-efficient synthesis of π-conjugated materials [30,32,33], in this study, we synthesized DPP-based molecules with A–D–A structures by using TDPP as the electron-deficient unit and phenyl derivative units as the electron-rich unit. Here, the α-C–H bonds on TDPP were directly arylated by the C–Br bonds from phenyl bromides under palladium catalysis. All of the target complex molecules were constructed from simple starting reactants and in a single step. The whole synthetic process was atom- and step-economical. The structures of nine molecules were characterized and verified by NMR, MALDI-TOF MS, and elemental analysis, and their optical and electrochemical properties were investigated by UV–vis absorption and cyclic voltammetry (CV) spectroscopy.

## 2. Results and Discussion

Firstly, TDPP and dibromobenzene derivatives were chosen as two kinds of substrates for the direct C–H arylation oligomerization. Here, the protocol of 1.5 mol % Pd_2_(dba)_3_ as pre-catalyst, 3 mol % P(*o*-MeOPh)_3_ as ligand, 30 mol % PivOH as additive, 2 equiv Cs_2_CO_3_ as base, and toluene as solvent, which were previously carefully developed by our group [33], was applied to these direct C–H arylated couplings. The carboxylic acid PivOH, employed herein, acted as a co-catalyst for the CMD activation of C–H bonds [34,35]. As can be seen in Table 1, by controlling the reactant ratio of the 1:0.4 between TDPP and dibromobenzene derivatives [33], the optimized yields of 50%~60% for the target molecules have been obtained. The α-C–H bonds on the starting TDPP were directly arylated by *m*-dibromobenzene derivatives, 1,3-dibromo-5-nitrobenzene, 1,3-dibromo-5-fluorobenzene, 1,3-dibromo-5-chlorobenzene, 1,3-dibromo-5-methoxy benzene, and 3,5-dibromo-1,1′-biphenyl, producing DPP-based molecules **Ms1~5**, respectively, with 50%, 57%, 53%, 49%, and 57% yields. The direct arylation coupling between DPP and the *p*-dibromobenzene derivatives, 1,4-dibromo-2,3-difluorobenzene, 1,4-dibromo-2,5-difluorobenzene, 1,4-dibromo-2,5-dichlorobenzene, and 1,4-dibromo-2,5-dimethoxy benzene, produced DPP-based molecules **Ms6~9**, respectively, with 50%, 61%, 56%, and 60% yields.

**Figure 1:**
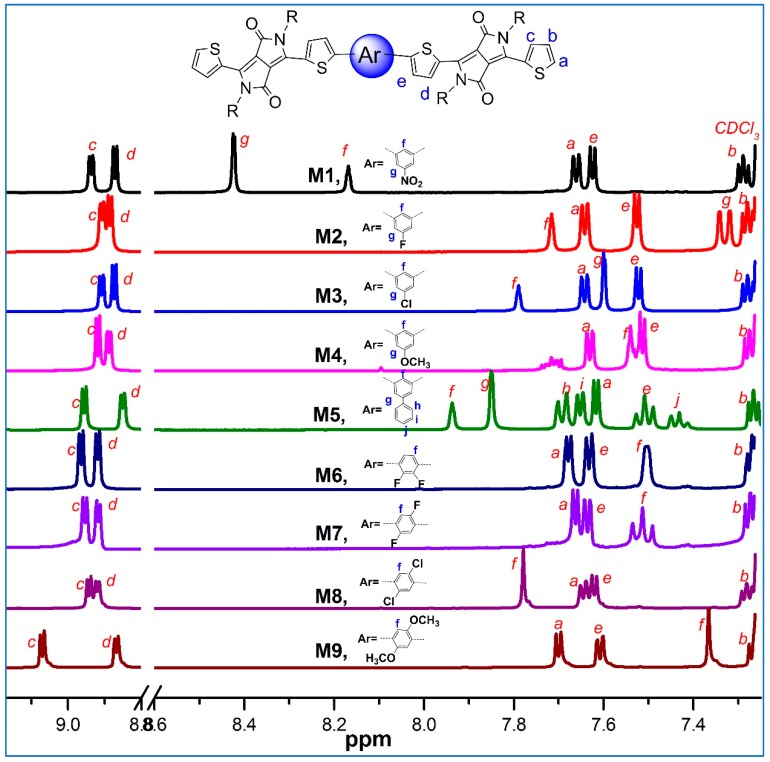
^1^H-NMR spectra for **Ms1~9** at 7.28–9.2 ppm.

All nine synthesized π-conjugated molecules have been characterized and verified by NMR, MALDI-TOF MS, and elemental analysis. The ^1^H-NMR analysis reveals spectra changes of molecules with phenyl cores bearing various groups, including electron-withdrawing groups such as –NO_2_, –F, and –Cl, electron-neutral phenyl groups, and the electron-donating group –OCH_3_. The electron-withdrawing keto-group on the DPP core has an effect on the neighboring aromatic hydrogens, causing their signals to shift downfield. As a result, all nine molecules have two sets of doublets in the downfield 8.65–9.31 ppm, which are assigned to the β-H on the thiophene rings linked to DPP core (Figure 1). The signals at ~4.0 ppm come from the –NCH_2_– group on the DPP cores, and the signals at 7.28–7.3 ppm should be assigned to β-hydrogen on the terminated thiophene rings of the molecule chains. The down- or upfield shifts of H*c* and H*d* of the molecules **Ms1~9** are ascribed to the phenyl cores bearing various electron-withdrawing or electron-donating groups. Figure 1 describes the detailed analysis and assignments of the aromatic hydrogen of all molecules.

The combined characterizations of ^1^H and ^13^C-NMR, MALDI-TOF MS and elemental analysis clearly demonstrates that the molecules **Ms1~9** have been successfully obtained. All the above spectra can be found in Supporting Information (SI). It is noteworthy that the nine synthesized molecules have good solubility in common organic solvents, such as CH_2_Cl_2_, toluene, CHCl_3_, and hexane, due to the presence of 2-hexyldecyl side chains. With these DPP-based D–A molecules **(Ms1~9)** in hand, we became interested in their molecular geometries and optical properties. The geometries of π-conjugated molecules were simulated by density functional theory (DFT) to understand the structure–property relationships. DFT calculations testified that the dihedral angle existed in the thiophene ring planes and phenyl ring planes (Figure S1). The molecules **Ms1~5** have torsion due to the *meta*-substitution of phenyl rings by DPP. The skeletons of the molecules **Ms1~5** exhibit a U-shaped appearance. DPP and benzene ring have a small torsion because of *para*-substitution of phenyl rings by DPP, strengthening the D–A interaction, which promotes the shift of π-electrons along the molecule backbones.

**Figure 2:**
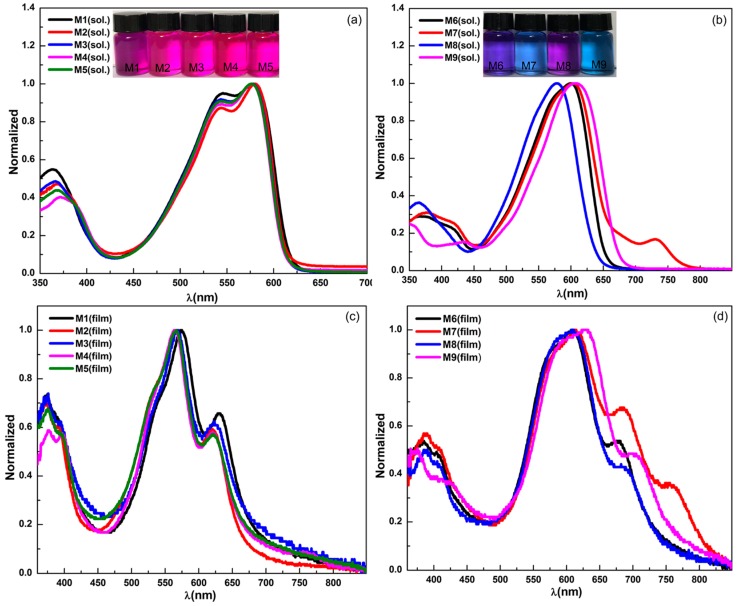
UV–vis spectra of *meta*-substituted phenyl-cored DPP molecules **Ms1~5** and *para*-substituted phenyl-cored DPP molecules **Ms6~9** (**b**) in CHCl_3_ (**a**,**b**) and in films (**c**,**d**).

As shown in Table 1, the phenyl cores of molecules bear various groups, including five electron-withdrawing groups, such as –NO_2_, –F, and –Cl, an aryl group, and electron-donating group –OCH_3_. These groups, with their different electronegativity, should influence the optical and electrochemical properties of the π-conjugated molecules. The optical properties of the molecules **Ms1~9** in solution and film were investigated using UV–vis spectroscopy. Figure 2 shows the UV–vis spectra of the molecules in CHCl_3_ solutions and films, and Table 2 summarizes the major optical properties, including light absorption peaks in CHCl_3_ solutions (λ_max_^s^) and solid films (λ_max_^f^) and corresponding absorption peak band-edges (λ_onset_^s^ and λ_onset_^f^). The molecules exhibited various colors in CHCl_3_, which corresponds to a wide range of light absorption between the visible and near-infrared regions. The light absorption peaks (λ_max_) and band-edges(λ_onset_) and the energy levels are summarized in Table 1. As can be seen in Figure 2a and Table 2, the substituent groups, –NO_2_, –F, –Cl, –OCH_3_, and phenyl, have little influence on the light absorptions of *meta*-phenyl-cored DPP molecules **Ms1~5**, which showed almost the same trend in UV–vis spectra with little differences in λ_max_s. The redshifts of λ_max_s from **M1** to **M2** and **M1** to **M3**, are 2 and −1.5 nm (Table 2), respectively, which mean electron-withdrawing nitro groups on the π-conjugated molecules should provide the molecules with higher electron affinities. The small differences in λ_max_s among **Ms1~5** should be attributed to the twisted geometries of *meta*-phenyl-cored DPP molecules, which caused a decreased conjugation and the disproportion between the repeating unit numbers and the effective conjugation lengths [36].

Compared with the *meta*-phenyl-cored DPP molecules **Ms1~5**, the substituent groups on the *para*-phenyl-cored DPP molecules **Ms6~9** have larger influences on UV–vis absorption. The λ_max_s of **Ms6~9** are 600, 603.5, 578.5, and 607 nm, respectively, which shows larger differences between each molecule and various corresponding colors exhibited by the CHCl_3_ solutions of molecules **Ms6~9** (Figure 2b). This might be due to the shortened conjugated length and the small contribution of groups in the *meta*-phenyl, thus, the enhanced delocalization of π-electron along the conjugated backbones and stronger D–A interaction between DPP and *para*-phenyl-cored molecules. The F and O atoms on **M6**, **M7**, and **M9** have F···H or O···H noncovalent interactions with the H atoms on thiophene rings [37]. Besides the O···H noncovalent interaction, the molecule **M9** also has *p*–π conjugation between the *p* orbital of O and the molecular backbone. As a result of synergic noncovalent interaction and *p*–π conjugation, **M9** possesses the longest wavelength of λ_max_ (607 nm, Table 2) among the nine molecules. For molecule **M8**, the larger size of Cl atom and its steric effect should cause higher torsion and dihedral angle between the phenyl ring and thiophene ring, and thus decrease the delocalization of π-electron along the molecule backbone. Consequently, molecule **M8** shows the shortest wavelength of λ_max_ among the *para*-phenyl-core molecules **Ms6~9**.

The UV–vis spectra for the solid films of molecules (Figure 2c,d) showed redshifts compared with their corresponding solutions because of their stronger intermolecular force, enhanced π–π stacking, and thus effective intermolecular charge transfer. The absorption band-edges (λ_onset_) of the π-conjugated molecules and the corresponding optical bandgaps (E_g_^opt^) calculated from E_g_^opt^ = 1240/λ_onset_ were also summarized in Table 2. The optical bandgaps of **Ms1~5** and **Ms6~9** are between 1.46 and 1.63 eV (Table 2).

**Figure 3:**
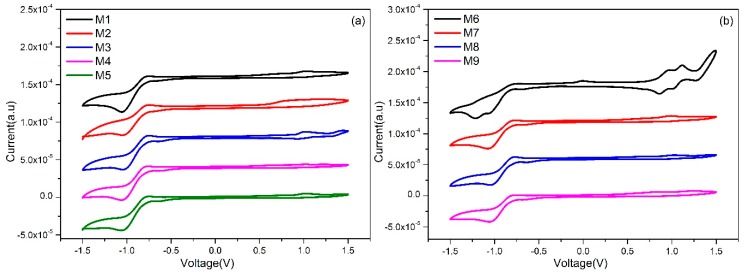
Cyclic voltammograms of *meta*-substitution phenyl-cored DPP molecules **Ms1~5** (**a**) and *para*-substitution phenyl-cored DPP molecules **Ms6~9** (**b**) in CH_2_Cl_2._

The electrochemical characters of **Ms1~9** were investigated by cyclic voltammetry (CV). The CV spectra of the nine DPPs are shown in Figure 3, and the measured frontier orbital energy (FMO) levels are summarized in Table 2. The lowest unoccupied molecular orbital (LUMO) energy levels were calculated based on the CV measurements, and the corresponding highest occupied molecular orbital (HOMO) levels were calculated from E_HOMO_ = E_LUMO_ − E_g_^opt^. The LUMO levels of all molecules generally decreased with the increase of electron-withdrawing ability of the substitutions on phenyl cores. According to above UV–vis and CV measurements, most of the molecules might be used in middle bandgap D–A conjugated materials for device applications. Particularly, molecule **M7** possesses deep HOMO levels and an extended absorption in the NIR region, which would promote its photoelectric properties for device applications.

## 3. Materials and Methods

### 3.1. Materials

Unless otherwise specified, all conventional chemicals were purchased from Energy Chemical (Shanghai, China). The starting TDPP was purchased from Derthon Co LTD (Shenzhen, China), 1,4-dibromo-2,5-difluorobenzene was purchased from SunaTech Inc (Suzhou, China). Anhydrous toluene was obtained by treating conventional toluene with CaH_2_.

### 3.2. Characterizations

All ^1^H and ^13^C-NMR spectra were obtained in chloroform-*d* or dichloromethane-*d* using a Bruker Avance 400 (^1^H-NMR 400MHz and ^13^C-NMR 101 MHz) spectrometer (Bruker, Germany). UV–vis absorption spectra were recorded on a Shimadzu UV-2450 spectrophotometer (Shimadzu Suzhou Instruments Mfg. Co. Ltd., Kyoto, Japan). Theoretical calculations based on DFT methods were performed for the molecules with Gaussian09 program Becke’s three-parameter gradient-corrected functional (B3LYP) with 6-31G(d,p) basis for geometric optimization. Cyclic voltammetry (CV) was done on a CHI 660E electrochemical workstation (Hua Ke Putian Technology Co. Ltd., Beijing, China) with Pt disk, Pt plate, and standard 10 calomel electrode (SCE) as working electrode, counter electrode, and reference electrode, respectively, in a 0.1 mol/L tetrabutylammonium hexafluorophosphate (Bu_4_NPF_6_) CH_2_Cl_2_ solution, and the obtained CV curves were calibrated by recording the ferrocene–ferrocenium (Fc/Fc^+^) redox couple (4.8 eV) below the vacuum level versus the potential of the SCE.

### 3.3. General Synthetic Procedure

Typically, TDPP (200 mg, 0.27 mmol), dibromobenzene derivatives 0.11 mmol, anhydrous Cs_2_CO_3_ (200 mg, 0.61 mmol), PivOH (7.9 mg, 0.08 mmol), Pd_2_(dba)_3_ (4.00 mg, 1.5 mol%), tris(*o*-methoxyphenyl) phosphine (3.08 mg, 3 mol%) were successively added into a Schlenk tube. The tube was purged by three repetitions of vacuum and argon filling. Then, 5 mL anhydrous toluene was added via syringe. The reaction solution was deoxygenated using three freeze–vacuum–thaw cycles, and then rigorously stirred at 100 °C for 24 h under argon atmosphere. Removal of the toluene by rotary evaporator afforded the crude product, which was then purified by chromatogragh column (CC) on silica gel using a mixture of CH_2_Cl_2_ and hexane as eluent, giving the target molecules **Ms1~9**.

### 3.4. Characterization Data of All Products

6′-(5,5′-(5-Nitro-1,3-phenylene)bis(thiophene-5,2-diyl))bis(2,5-bis(2-hexyldecyl)-3-(thiophen-2-yl)pyrrolo[3,4-c]pyrrole-1,4(2H,5H)-dione) (**M1**): 95.53 mg, 50% yield. ^1^H-NMR (400 MHz, CDCl_3_) δ 8.91 (dd, *J* = 26.3, 3.9 Hz, 4H), 8.43 (d, *J* = 1.3 Hz, 2H), 8.17 (s, 1H), 7.65 (dd, *J* = 14.8, 4.5 Hz, 4H), 7.29 (dd, *J* = 9.0, 4.2 Hz, 2H), 4.06 (dd, *J* = 12.2, 7.8 Hz, 8H), 2.07–1.87 (m, 4H), 1.51–1.01 (m, 96H), 0.83 (dd, *J* = 18.3, 5.5 Hz, 24H).^13^C-NMR (101 MHz, CDCl_3_) δ 161.81, 161.53, 149.45, 144.96, 141.37, 138.83, 135.92, 135.76, 131.01, 129.72, 128.53, 126.41, 120.05, 109.16, 108.03, 77.33, 77.02, 76.70, 46.34, 37.98, 37.76, 31.88, 31.85, 31.76, 31.34, 31.22, 30.04, 30.01, 29.70, 29.67, 29.52, 29.50, 29.29, 26.33, 22.66, 22.62, 14.10, 14.07, 14.03.MALDI-TOF MS (*m*/*z*): [M]^+^ calcd for C_98_H_145_N_5_O_6_S_4_: 1617.5070, found 1617.0710. Elemental analysis: calcd for C_98_H_145_N_5_O_6_S_4_, C, 72.77; H, 9.04; N, 4.33%. Found: C, 72.74; H, 9.07; N, 4.35%.

6,6′-(5,5′-(5-fluoro-1,3-phenylene)bis(thiophene-5,2-diyl))bis(2,5-bis(2-hexyldecyl)-3-(thiophen-2-yl)pyrrolo[3,4-c]pyrrole-1,4(2H,5H)-dione) (**M2**): 103.0 mg, 56.7% yield. ^1^H-NMR (400 MHz, CDCl_3_) δ 8.90 (dd, *J* = 8.7, 3.8 Hz, 4H), 7.72 (s, 1H), 7.64 (d, *J* = 4.9 Hz, 2H), 7.53 (d, *J* = 4.0 Hz, 2H), 7.33 (d, *J* = 9.0 Hz, 2H), 7.31–7.27 (m, 2H), 4.05 (s, 8H), 1.94 (s, 4H), 1.22 (s, 96H), 0.99–0.74 (m, 24H).^13^C-NMR (101 MHz, CDCl_3_) δ 161.75, 146.79, 140.80, 136.18, 135.46, 130.72, 130.02, 129.82, 128.46, 125.61, 108.73, 77.33, 77.01, 76.69, 46.31, 37.95, 37.76, 31.88, 31.84, 31.79, 31.76, 31.36, 31.23, 30.05, 30.01, 29.71, 29.66, 29.54, 29.49, 29.29, 26.35, 26.23, 22.62, 14.09, 14.06, 14.02. MALDI-TOF MS (*m*/*z*): [M]^+^ calcd for C_98_H_145_FN_4_O_4_S_4_: 1590.5004, found 1589.9700. Elemental analysis: calcd for C_98_H_145_FN_4_O_4_S_4_, C, 74.01; H, 9.19; N, 3.52%. Found: C, 74.07; H, 9.22; N, 3.50%.

6,6′-(5,5′-(5-chloro-1,3-phenylene)bis(thiophene-5,2-diyl))bis(2,5-bis(2-hexyldecyl)-3-(thiophen-2-yl)pyrrolo[3,4-c]pyrrole-1,4(2H,5H)-dione) (**M3**): 97.69 mg, 53.1% yield. ^1^H-NMR (400 MHz, CDCl_3_) δ 9.23–8.65 (m, 4H), 7.79 (s, 1H), 7.74–7.62 (m, 2H), 7.56 (dd, *J* = 30.7, 2.7 Hz, 4H), 7.31 (dd, *J* = 26.6, 22.6 Hz, 2H), 4.33–3.73 (m, 8H), 1.97 (t, *J* = 16.0 Hz, 4H), 1.50–1.05 (m, 96H), 0.98–0.72 (m, 24H). ^13^C-NMR (101 MHz, CDCl_3_) δ 161.75, 161.68, 146.51, 140.82, 139.47, 136.14, 135.77, 135.48, 130.75, 130.12, 129.81, 128.47, 125.64, 108.73, 77.33, 77.01, 76.70, 46.31, 37.95, 37.75, 31.88, 31.85, 31.80, 31.76, 31.23, 30.06, 30.01, 29.30, 26.40, 26.23, 26.19, 22.63, 14.08, −0.01. MALDI-TOF MS (*m*/*z*): [M]^+^ calcd for C_98_H_145_ClN_4_O_4_S_4_: 1606.9520, found 1606.0140. Elemental analysis: calcd for C_98_H_145_ClN_4_O_4_S_4_, C, 73.25; H, 9.10; N, 3.49%. Found: C, 73.26; H, 9.17; N, 3.53%.

6,6′-(5,5′-(5-methoxy-1,3-phenylene)bis(thiophene-5,2-diyl))bis(2,5-bis(2-hexyldecyl)-3-(thiophen-2-yl)pyrrolo[3,4-c]pyrrole-1,4(2H,5H)-dione) (**M4**): 90.80 mg, 49.5% yield. ^1^H-NMR (400 MHz, CDCl_3_) δ 8.91 (dd, *J* = 12.5, 4.0 Hz, 4H), 7.63 (d, *J* = 5.9 Hz, 2H), 7.57–7.45 (m, 3H), 7.28 (d, *J* = 4.9 Hz, 2H), 7.17 (s, 2H), 4.09–3.93 (m, 8H), 1.96 (s, 4H), 1.52–1.00 (m, 96H), 0.94–0.76 (m, 26H). ^13^C-NMR (101 MHz, CDCl_3_) δ 161.73, 148.43, 140.43, 139.94, 136.44, 135.41, 135.26, 129.88, 129.41, 128.42, 125.20, 108.42, 77.33, 77.01, 76.69, 55.60, 46.31, 37.92, 37.76, 31.88, 31.79, 31.76, 31.35, 31.23, 30.39, 30.07, 30.01, 29.73, 29.54, 29.50, 29.29, 28.94, 27.74, 26.36, 26.20, 23.78, 22.98, 22.62, 19.16, 14.07, 14.03.MALDI-TOF MS (*m*/*z*): [M]^+^ calcd for C_99_H_148_N_4_O_5_S_4_: 1602.5360, found 1602.0710. Elemental analysis: calcd for C_99_H_148_N_4_O_5_S_4_, C, 74.20; H, 9.31; N, 3.50%. Found: C, 74.59; H, 9.34; N, 3.46%.

6,6′-(5,5′-([1,1′-biphenyl]-3,5-diyl)bis(thiophene-5,2-diyl))bis(2,5-bis(2-hexyldecyl)-3-(thiophen-2-yl)pyrrolo[3,4-c]pyrrole-1,4(2H,5H)-dione) (**M5**): 126.09 mg, 57.3% yield. ^1^H-NMR (400 MHz, CD_2_Cl_2_) δ 8.98 (d, *J* = 4.1 Hz, 2H), 8.87 (d, *J* = 3.7 Hz, 2H), 7.96 (s, 1H), 7.87 (s, 2H), 7.71 (d, *J* = 7.4 Hz, 2H), 7.67 (d, *J* = 4.9 Hz, 2H), 7.64 (d, *J* = 4.1 Hz, 2H), 7.53 (t, *J* = 7.5 Hz, 2H), 7.45 (t, *J* = 7.3 Hz, 1H), 7.32–7.25 (m, 2H), 4.04 (dd, *J* = 17.3, 7.6 Hz, 8H), 1.93 (d, *J* = 26.9 Hz, 4H), 1.33–0.99 (m, 96H), 0.88–0.76 (m, 24H). ^13^C-NMR (101 MHz, CD_2_Cl_2_) δ 161.57, 139.69, 136.38, 134.95, 129.02, 128.22, 127.18, 125.12, 68.33, 53.94, 53.67, 53.40, 53.13, 52.86, 46.11, 37.96, 37.77, 31.88, 31.84, 31.76, 31.31, 31.19, 30.04, 29.98, 29.65, 29.50, 29.30, 26.29, 26.16, 22.63, 13.84. MALDI-TOF MS (*m*/*z*): [M]^+^ calcd for C_104_H_150_N_4_O_4_S_4_: 1648.6080, found 1648.3150. Elemental analysis: calcd for C_104_H_150_N_4_O_4_S_4_, C, 75.77; H, 9.17; N, 3.40%. Found: C, 75.88; H, 9.22; N, 3.51%.

6,6′-(5,5′-(2,3-difluoro-1,4-phenylene)bis(thiophene-5,2-diyl))bis(2,5-bis(2-hexyldecyl)-3-(thiophen-2-yl)pyrrolo[3,4-c]pyrrole-1,4(2H,5H)-dione) (**M6**): 92.15 mg, 50.1% yield. ^1^H-NMR (400 MHz, CDCl_3_) δ 8.94 (dd, *J* = 18.1, 4.0 Hz, 4H), 7.68 (d, *J* = 4.1 Hz, 2H), 7.64 (d, *J* = 5.0 Hz, 2H), 7.50 (s, 2H), 7.28 (d, *J* = 3.9 Hz, 2H), 4.05 (dd, *J* = 12.8, 7.7 Hz, 8H), 1.95 (s, 4H), 1.51–1.07 (m, 96H), 1.00–0.70 (m, 24H). ^13^C-NMR (101 MHz, CDCl_3_) δ 161.65, 140.78, 140.18, 139.44, 135.89, 135.54, 130.76, 129.81, 128.47, 127.99, 122.73, 108.84, 108.14, 77.33, 77.02, 76.70, 46.32, 38.05, 37.77, 31.88, 31.81, 31.76, 31.35, 31.22, 30.05, 30.02, 29.73, 29.68, 29.54, 29.50, 29.30, 26.33, 26.22, 22.66, 22.63, 14.09, 14.07, −0.01.MALDI-TOF MS (*m*/*z*): [M]^+^ calcd for C_98_H_144_F_2_N_4_O_4_S_4_: 1608.4900, found 1608.2780. Elemental analysis: calcd for C_98_H_144_F_2_N_4_O_4_S, C, 73.18; H, 9.02; N, 3.48%. Found: C, 73.25; H, 9.08; N, 3.52%.

6,6′-(5,5′-(2,5-difluoro-1,4-phenylene)bis(thiophene-5,2-diyl))bis(2,5-bis(2-hexyldecyl)-3-(thiophen-2-yl)pyrrolo[3,4-c]pyrrole-1,4(2H,5H)-dione) (**M7**): 80.36 mg, 60.6% yield. ^1^H-NMR (400 MHz, CDCl_3_) δ 8.94 (dd, *J* = 14.9, 4.0 Hz, 4H), 7.65 (dd, *J* = 10.8, 4.3 Hz, 4H), 7.52 (t, *J* = 8.9 Hz, 2H), 7.28 (d, *J* = 4.8 Hz, 2H), 4.05 (dd, *J* = 12.4, 7.7 Hz, 8H), 1.94 (s, 4H), 1.49–1.05 (m, 96H), 0.85 (dd, *J* = 13.4, 6.7 Hz, 24H). ^13^C-NMR (101 MHz, CDCl_3_) δ 161.67, 139.45, 135.55, 130.78, 129.81, 128.48, 108.17, 100.72, 77.33, 77.01, 76.69, 46.34, 38.05, 37.76, 31.88, 31.79, 31.76, 31.35, 31.22, 30.05, 30.01, 29.72, 29.50, 29.29, 26.33, 26.19, 22.66, 22.62, 14.09, 14.06. MALDI-TOF MS (*m*/*z*): [M]^+^ calcd for C_98_H_144_F_2_N_4_O_4_S_4_: 1608.4700, found 1608.6640. Elemental analysis: calcd for C_98_H_144_F_2_N_4_O_4_S, C, 73.18; H, 9.02; N, 3.48%. Found: C, 73.29; H, 9.09; N, 3.52%.

6,6′-(5,5′-(2,5-dichloro-1,4-phenylene)bis(thiophene-5,2-diyl))bis(2,5-bis(2-hexyldecyl)-3-(thiophen-2-yl)pyrrolo[3,4-c]pyrrole-1,4(2H,5H)-dione) (**M8**): 104.87 mg, 55.8% yield. ^1^H-NMR (400 MHz, CDCl_3_) δ 8.86 (dd, *J* = 8.9, 4.0 Hz, 4H), 7.71 (s, 2H), 7.69–7.23 (m, 4H), 7.21 (s, 2H), 3.97 (d, *J* = 7.8 Hz, 8H), 1.88 (dd, *J* = 12.2, 5.4 Hz, 4H), 1.40–0.97 (m, 96H), 0.89–0.70 (m, 24H). ^13^C-NMR (101 MHz, CDCl_3_) δ 160.77, 160.66, 141.64, 139.88, 138.51, 134.52, 134.24, 131.68, 131.38, 130.44, 129.77, 129.66, 128.79, 128.51, 127.46, 107.81, 107.11, 76.32, 76.00, 75.68, 45.31, 37.06, 36.75, 30.87, 30.77, 30.31, 30.22, 29.05, 29.00, 28.69, 28.66, 28.51, 28.28, 25.32, 25.21, 25.18, 21.65, 21.62, 13.08, 13.06, 0.00, −1.03.MALDI-TOF MS (*m*/*z*): [M]^+^ calcd for C_98_H_144_Cl_2_N_4_O_4_S_4_: 1641.3800, found 1641.6190. Elemental analysis: calcd for C_98_H_144_Cl_2_N_4_O_4_S, C, 71.71; H, 8.84; N, 3.41%. Found: C, 71.78; H, 8.92; N, 3.47%.

6,6′-(5,5′-(2,5-dimethoxy-1,4-phenylene)bis(thiophene-5,2-diyl))bis(2,5-bis(2-hexyldecyl)-3-(thiophen-2-yl)pyrrolo[3,4-c]pyrrole-1,4(2H,5H)-dione) (**M9**): 80.80 mg, 59.7% yield. ^1^H-NMR (400 MHz, CDCl_3_) δ 9.00 (d, *J* = 4.2 Hz, 2H), 8.80 (d, *J* = 3.8 Hz, 2H), 7.58 (dd, *J* = 37.2, 4.7 Hz, 4H), 7.45–7.19 (m, 4H), 4.04 (d, *J* = 7.5 Hz, 6H), 3.97 (d, *J* = 4.1 Hz, 8H), 1.90 (d, *J* = 39.8 Hz, 4H), 1.40–0.97 (m, 96H), 0.91–0.63 (m, 24H). ^13^C-NMR (101 MHz, CDCl_3_) δ 161.69, 150.37, 139.70, 135.86, 130.20, 126.40, 111.27, 108.37, 77.32, 77.01, 76.69, 56.40, 31.88, 31.76, 31.24, 30.12, 29.68, 29.51, 29.30, 26.21, 22.64, 14.08, 1.02. MALDI-TOF MS (*m*/*z*): [M]^+^ calcd for C_100_H_150_N_4_O_6_S_4_:1632.5400, found 1632.5570. Elemental analysis: calcd for C_100_H_150_N_4_O_6_S_4_, C, 73.57; H, 9.26; N, 3.43%. Found: C, 73.59; H, 9.27; N, 3.48%.

## 4. Conclusions

In summary, direct arylation of the C–H bond has been demonstrated as an atom- and step-economic synthetic strategy for accessing DPP-based molecules. Nine DPP-based novel molecules **Ms1~9**, including five *meta*-phenyl-cored DPP molecules and four *para*-phenyl-cored DPP molecules, have all been synthesized in one step, in a facile manner, via direct C–H arylation. All nine conjugated molecules, which had D–A structures and middle optical bands, have been well-characterized by NMR, MALDI-TOF MS, elemental analysis, cyclic voltammetry, and UV–vis absorption spectra. The current study would be a good reference for the atom- and step-economic synthesis of large DPP-based conjugated molecules.

## Figures and Tables

**Table 1:** One step synthesis of phenyl-cored DPP molecules **(Ms1~9)** ^1,2^.

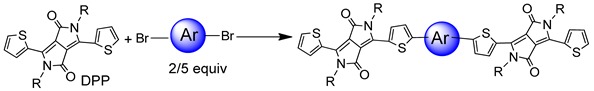
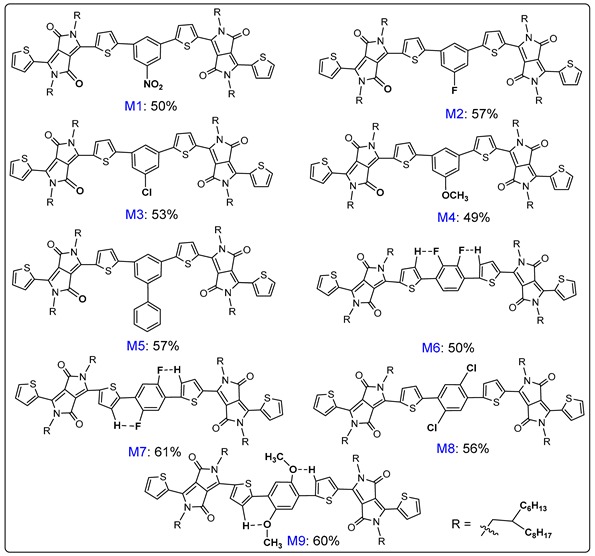

^1^ All the reactions were conducted with TDPP (200 mg, 0.27 mmol, 1 equiv), dibromoarene R’ArBr_2_ (2/5 equiv), PivOH (7.9 mg, 0.08 mmol), anhydrous Cs_2_CO_3_ (200 mg, 0.61 mmol), Pd_2_(dba)_3_ (4.0 mg, 1.5 mol %), and P(*o*-MeOPh)_3_ (3.08 mg, 3 mol %) in 5 mL toluene at 100 °C for 24 h under argon atmosphere. ^2^ Isolated yields.

**Table 2:** Optical and electrochemical properties of molecules **Ms1~9**.

Molecules	λ_max_^s^ (nm)	λ_max_^f^ (nm)	LUMO (eV)	λ_onset_^s^ (nm)	λ_onset_^f^ (nm)	E_g_^opt^ (eV)	HOMO (eV)
**M**1	578.0	575.0	−3.94	649	800	1.55	−5.49
**M**2	580.0	565.5	−3.99	649	760	1.63	−5.62
**M**3	576.5	567.5	−3.95	649	800	1.55	−5.50
**M**4	576.5	565.5	−3.94	649	800	1.55	−5.49
**M**5	576.5	566.5	−3.95	649	800	1.55	−5.50
**M**6	600.0	609.5	−3.93	685	850	1.46	−5.39
**M**7	603.5	609.5	−3.95	800	790	1.57	−5.52
**M**8	578.5	611.0	−3.97	632	850	1.46	−5.43
**M**9	607.0	629.5	−3.95	700	850	1.46	−5.41

## Supplementary Materials

The following are available online at https://www.mdpi.com/1420-3049/24/9/1760/s1. Theoretical calculations based on DFT methods have been performed for the oligomers with Gaussian09 program: Figure S1; copies of ^1^H-NMR and ^13^C-NMR spectra of the products: Figures S2–S10; mass spectra of all oligomers: Figures S11–S19.
