# Supplementary material for: Novel Diketopyrrolopyrrole-Based π-Conjugated Molecules Synthesized Via One-Pot Direct Arylation Reaction
Source: Molecules. 2019 May 7;24(9):1760. doi: 10.3390/molecules24091760 (PMC6539255; doi:10.3390/molecules24091760)
Supplement: Supplementary file 1 [file molecules-24-01760-s001.pdf]

# Novel Diketopyrrolopyrrole-based π-Conjugated Molecules Synthesized *via* One-Pot Direct Arylation Reaction

**Hui Liu [a] Xiao-Feng Zhang [a] Jing-Zhao Cheng [a] Ai-Guo Zhong[b] He-Rui Wen*,[a] and Shi-Yong Liu*,[a]**

[a]*School of Metallurgical and Chemical Engineering, Jiangxi University of Science and Technology, Ganzhou 341000, China*
[b]*School of Materials and Metallurgy, Inner Mongolia University of Science and Technology, Baotou, 014010, China*

## Supplementary Information

### Contents

1. DFT-optimized geometries.................................................. S2

2. $^{1}H$ and $^{13}C$ NMR.................................................. S3-11

3. Maldi-TOF MS spectra.................................................. S12-20

# 1.DFT-optimized geometries

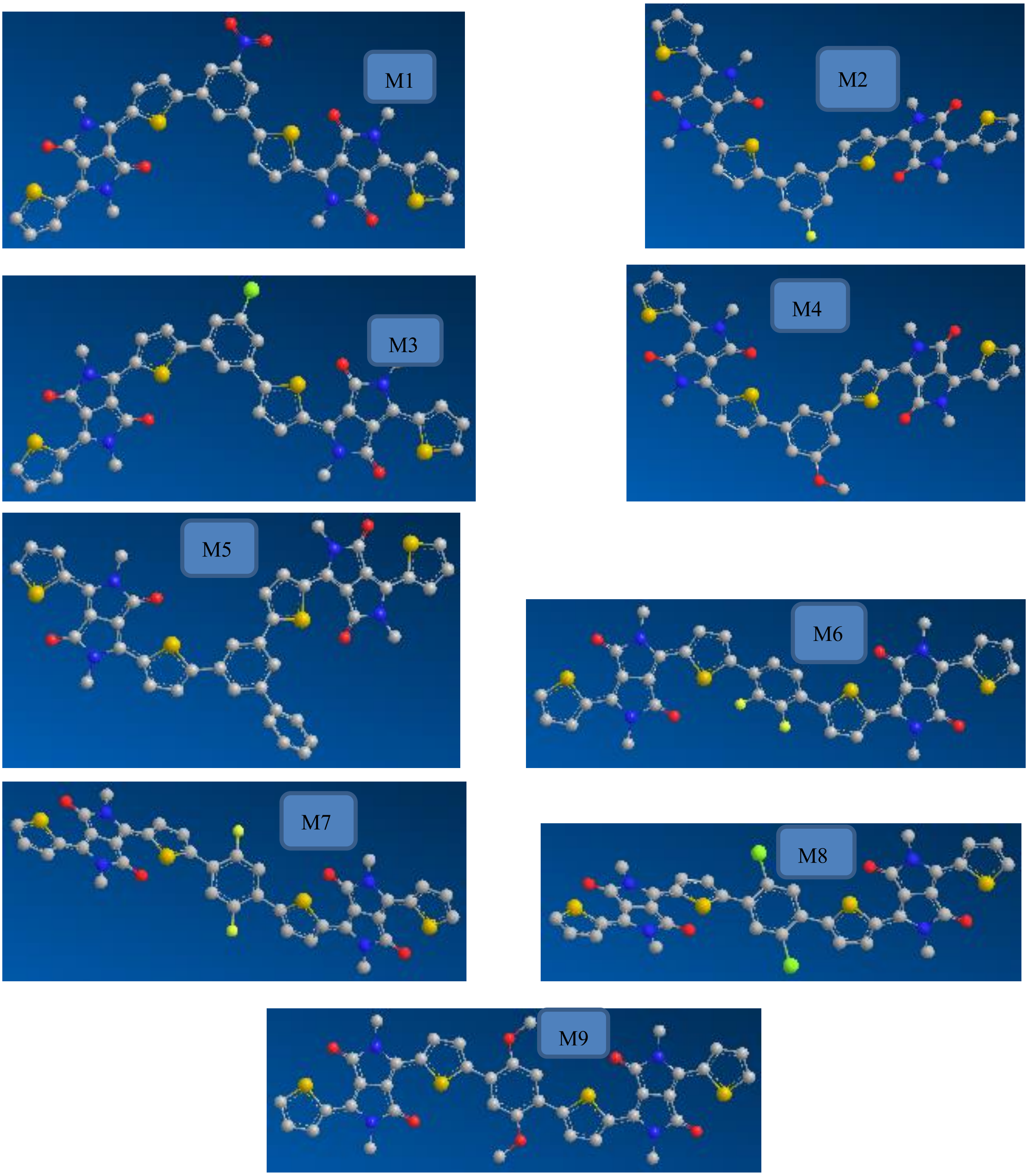


**Figure S1** DFT-optimized geometries of Molecules **Ms1~9** (the alkyl chains replaced by methyl groups).

# 2. $^{1}$H and $^{13}$C NMR spectra

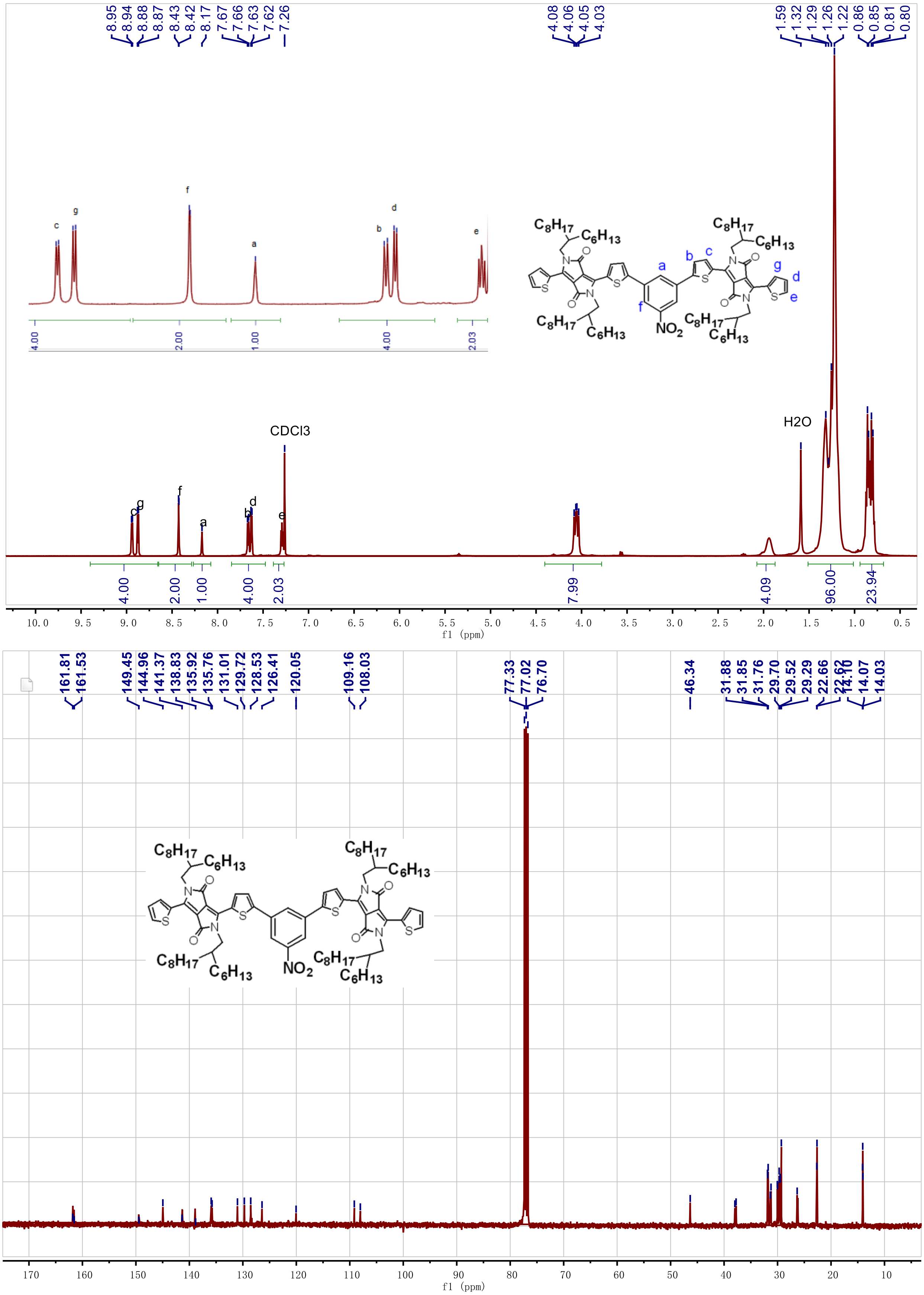


**Figure S2** $^{1}$H and $^{13}$C NMR spectra of **M1** in $CDCl_3$.

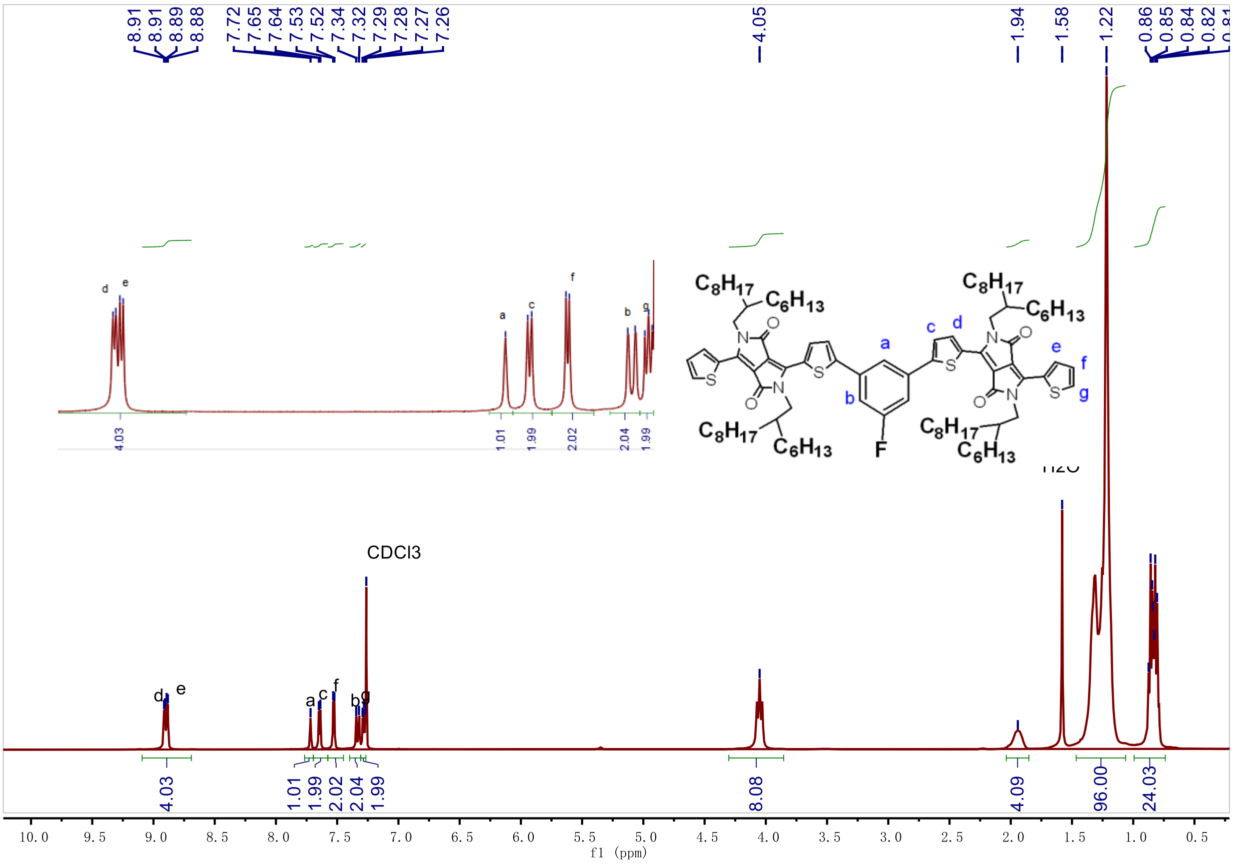

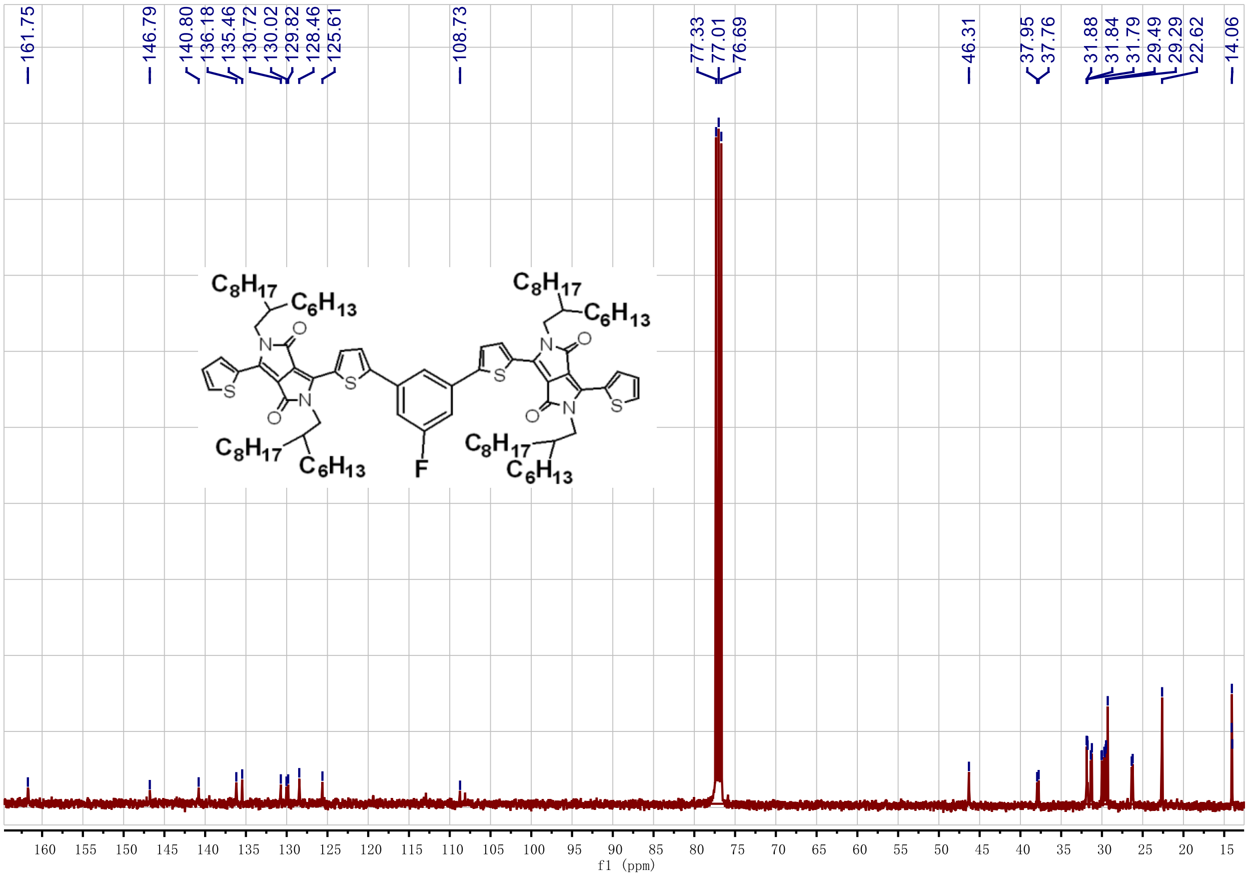

**Figure S3** $^{1}$H and $^{13}$C NMR spectra of **M2** in $CDCl_3$.

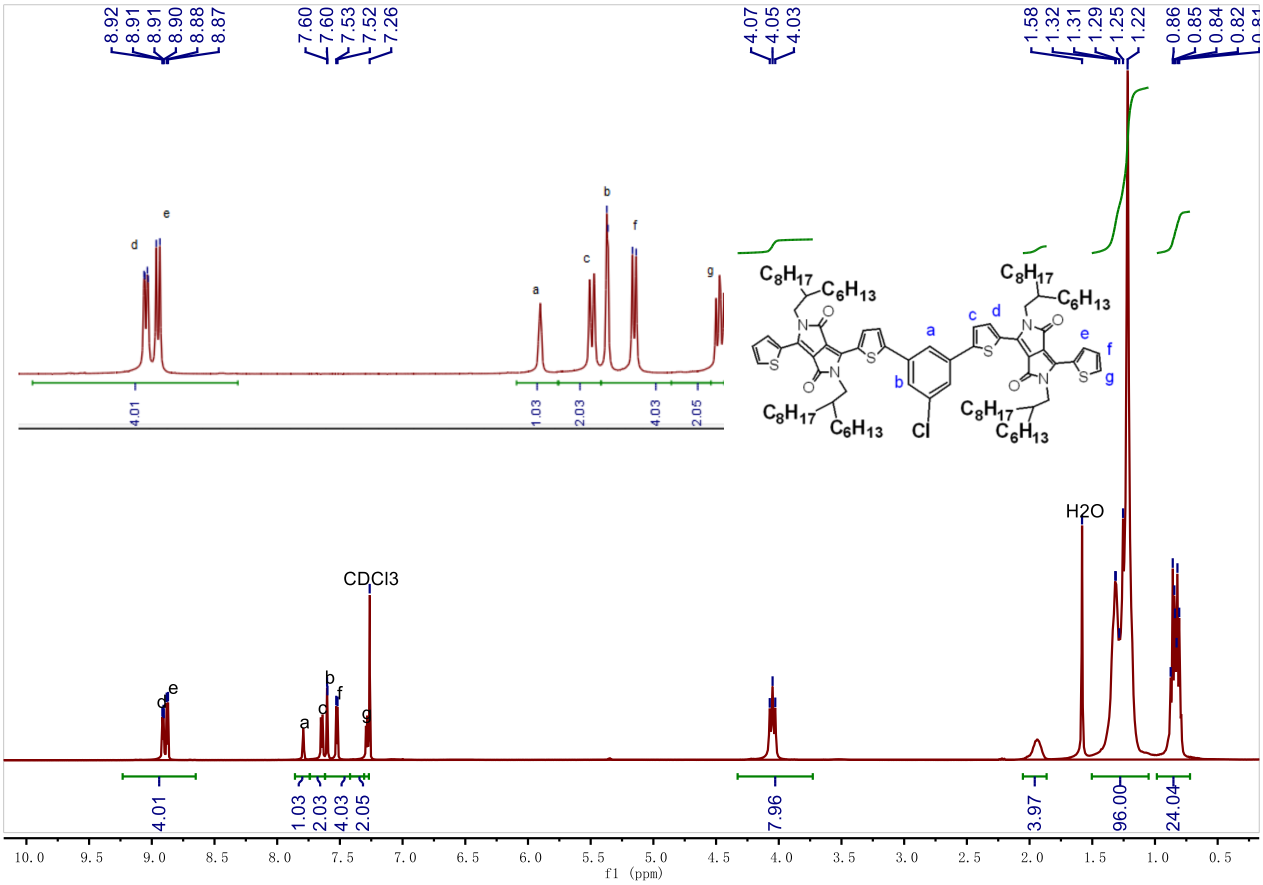

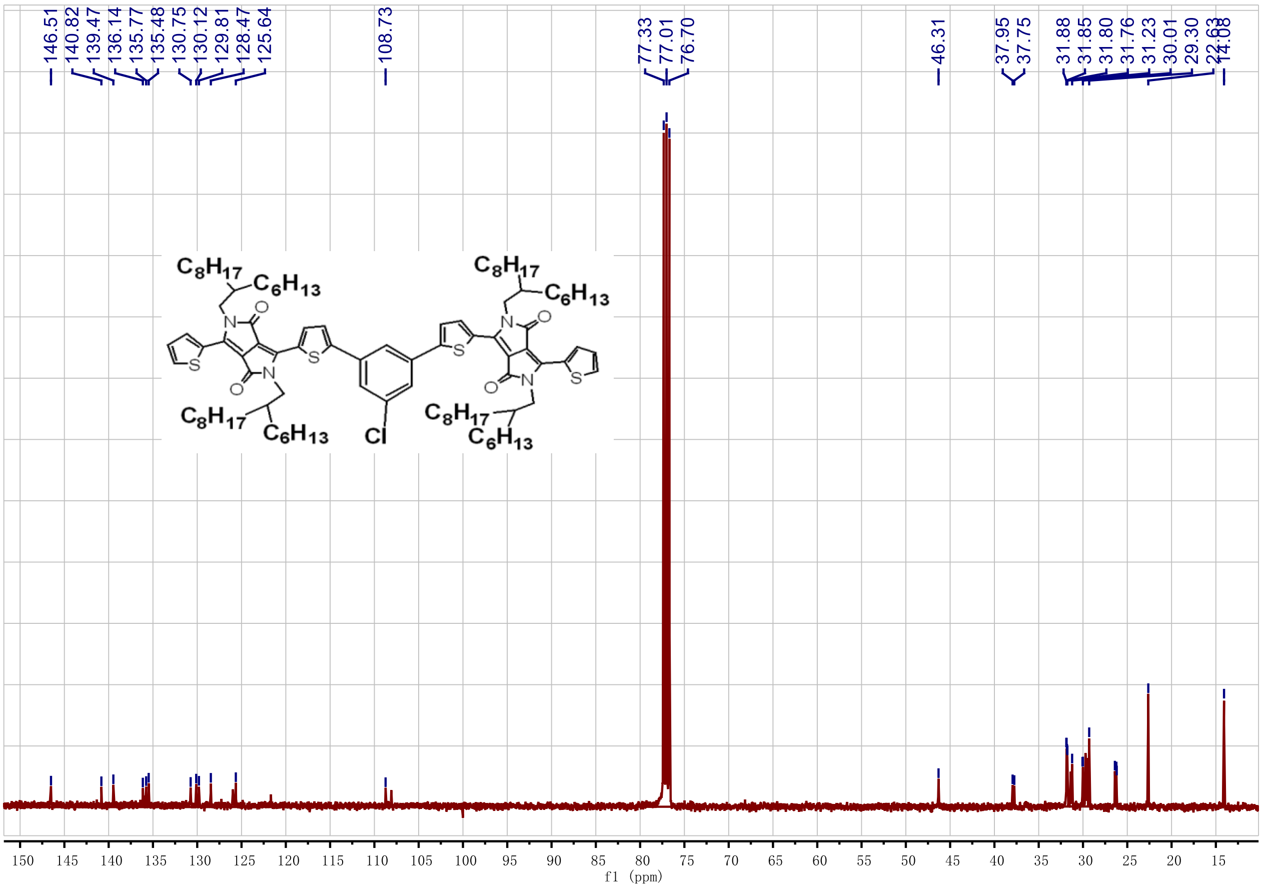

**FigureS4** $^{1}H$ and $^{13}C$ NMR spectra of **M**3 in $CDCl_3$.

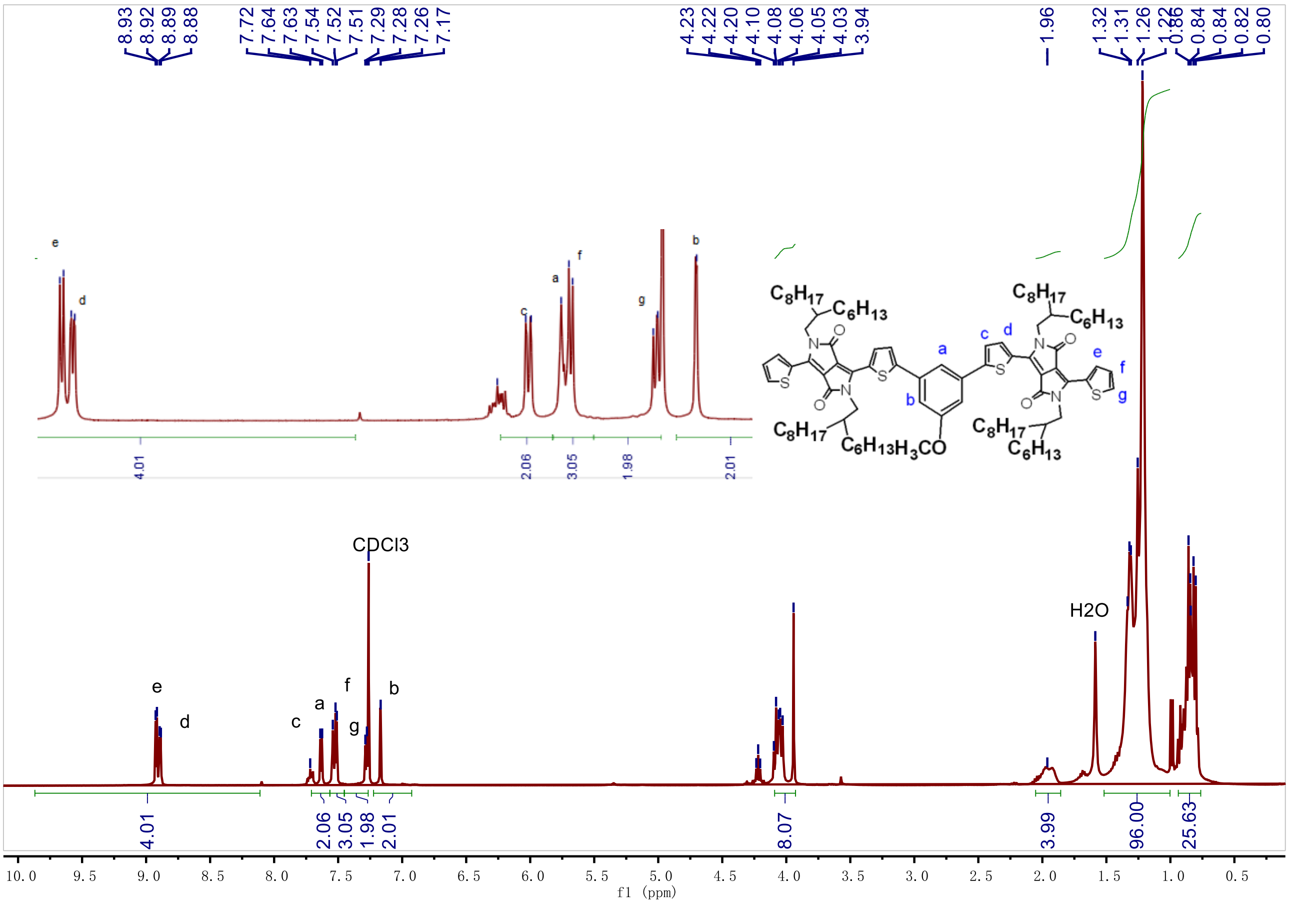


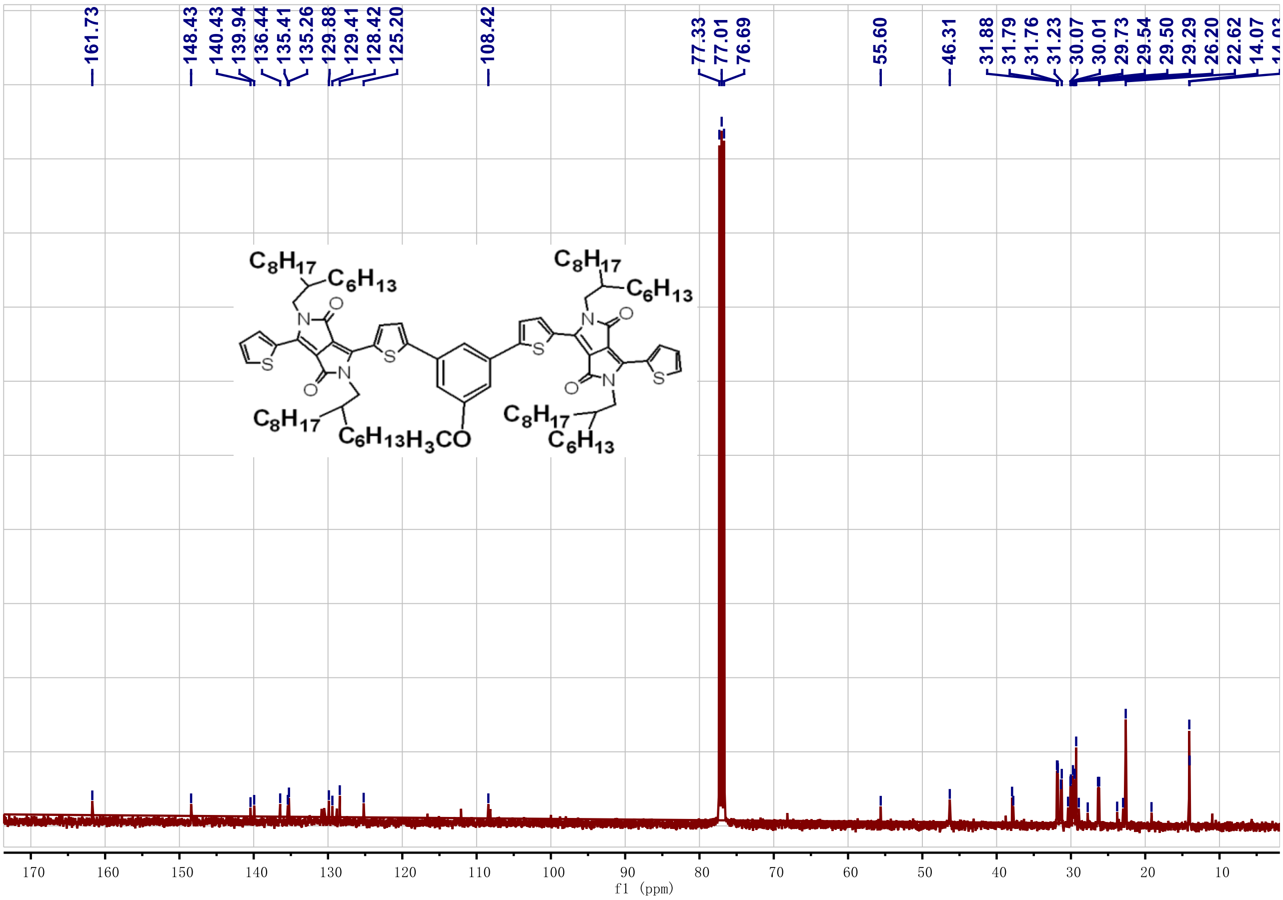


**Figure S5** $^{1}$H and $^{13}$C NMR spectra of **M4** in $CDCl_3$.

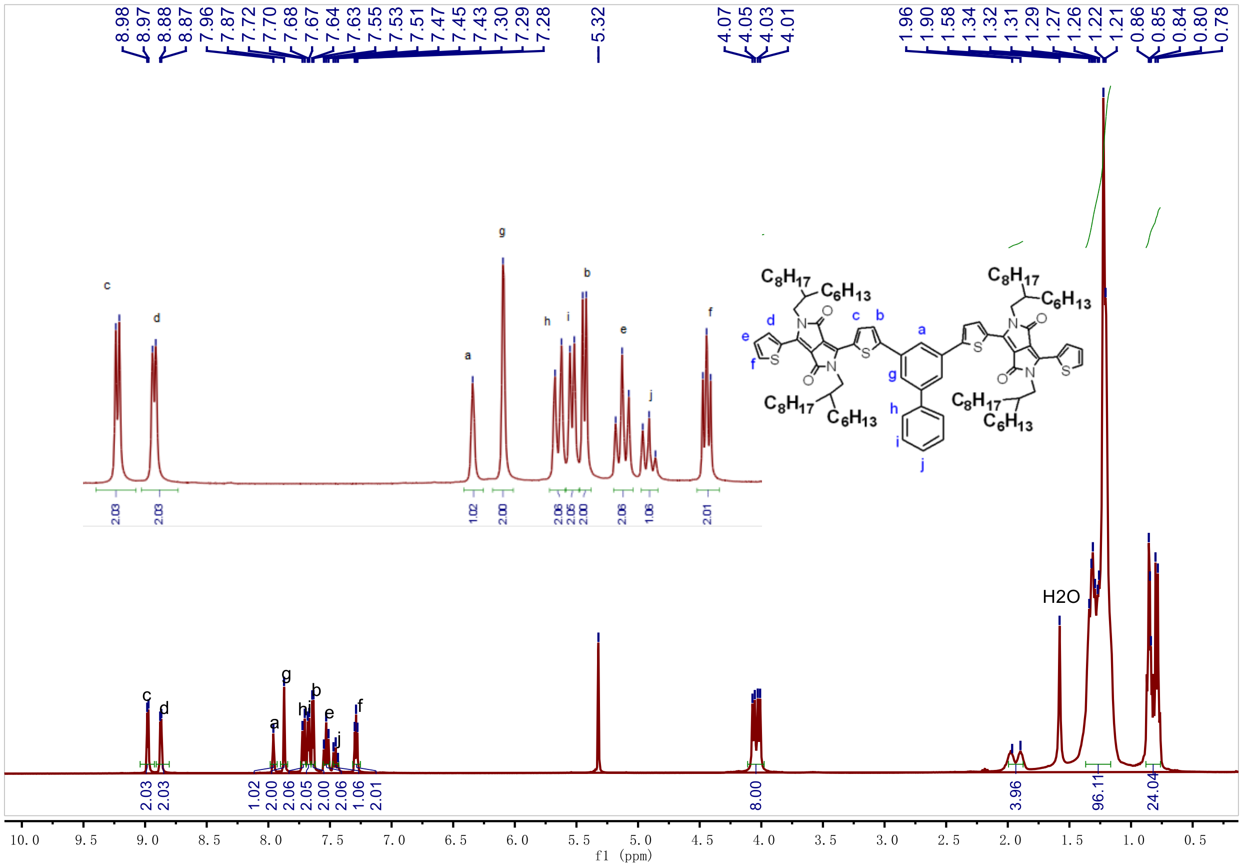

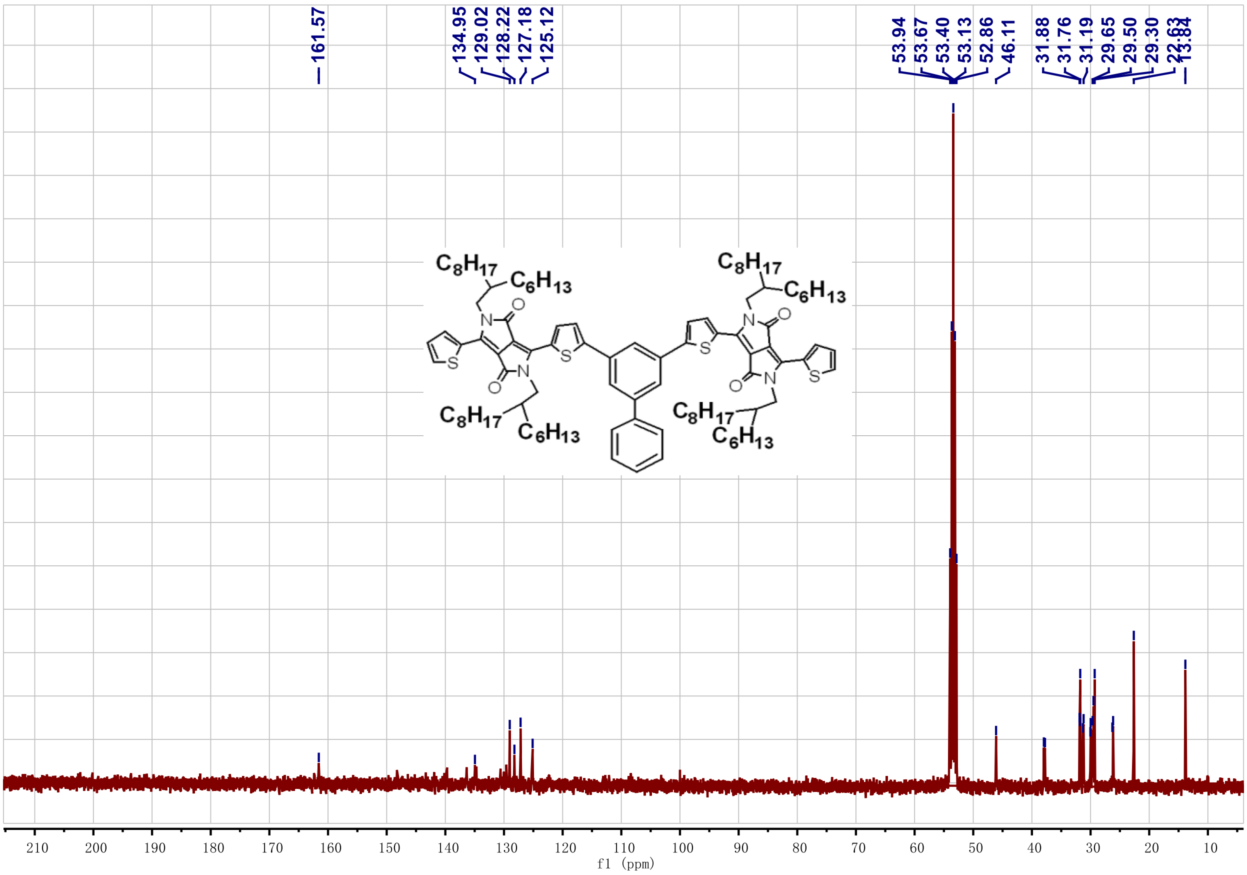

**Figure S6** $^{1}H$ and $^{13}C$ NMR spectra of **M5** in $CDCl_3$.

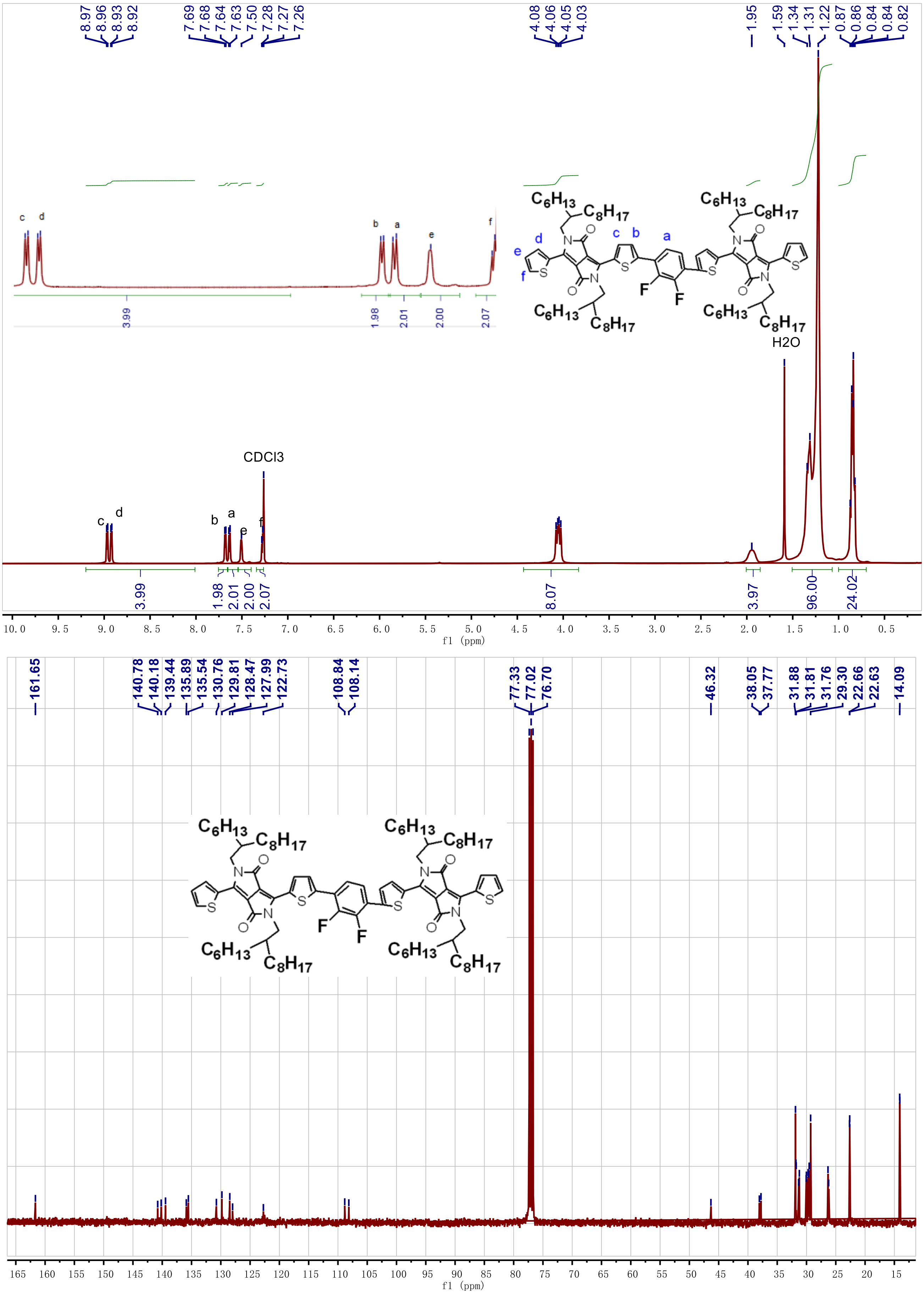


**Figure S7** $^{1}$H and $^{13}$C NMR spectra of **M6** in $CDCl_3$.

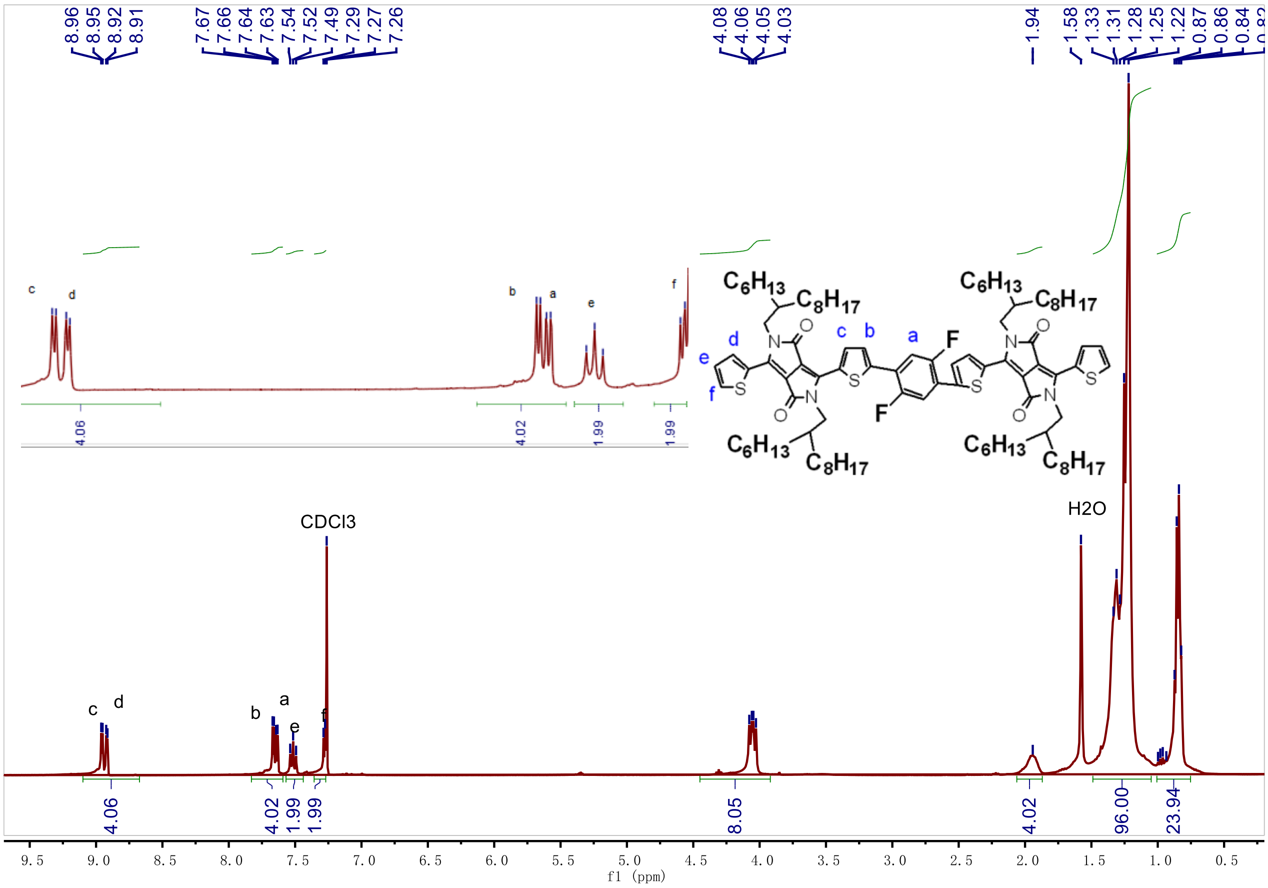

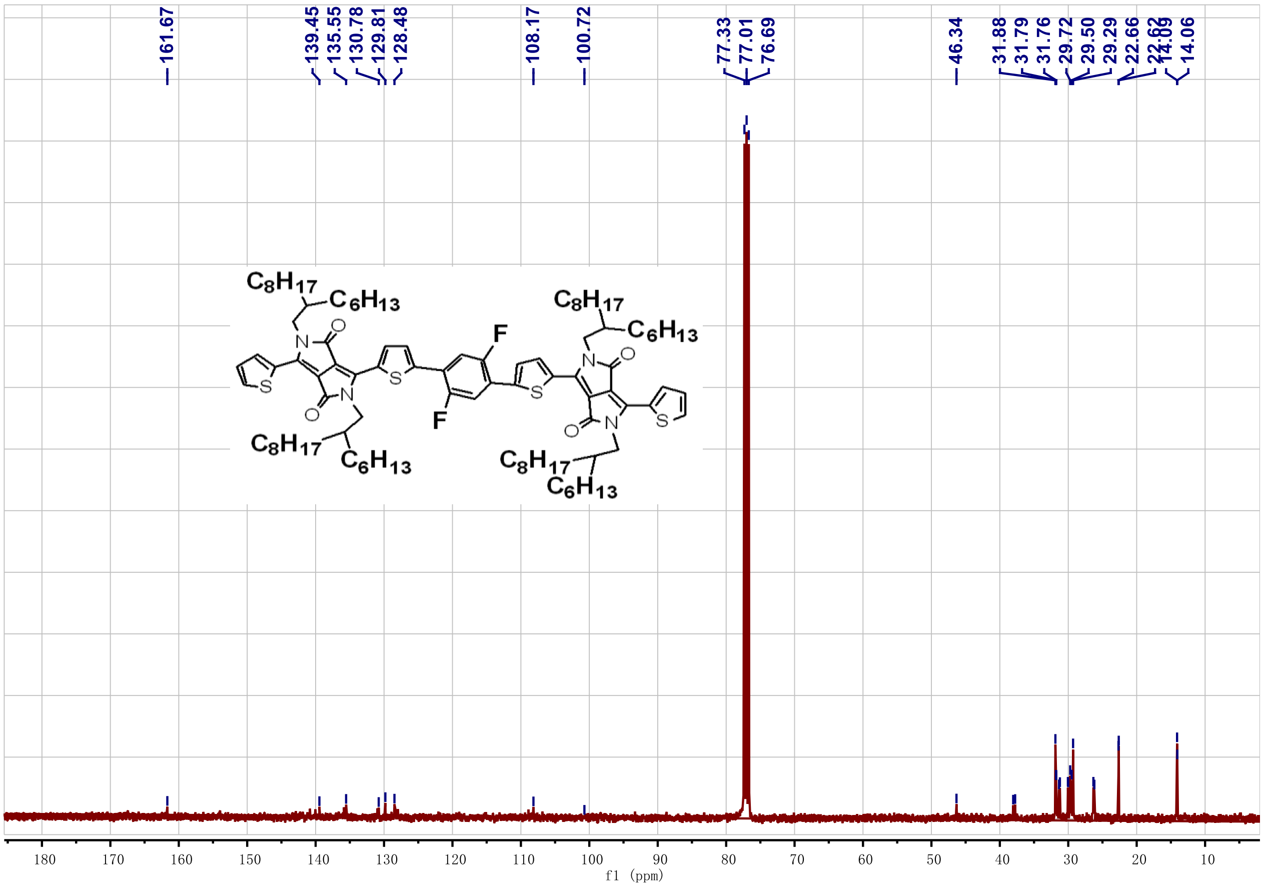

**Figure S8** $^{1}$H and $^{13}$C NMR spectra of **M7** in $CDCl_3$.

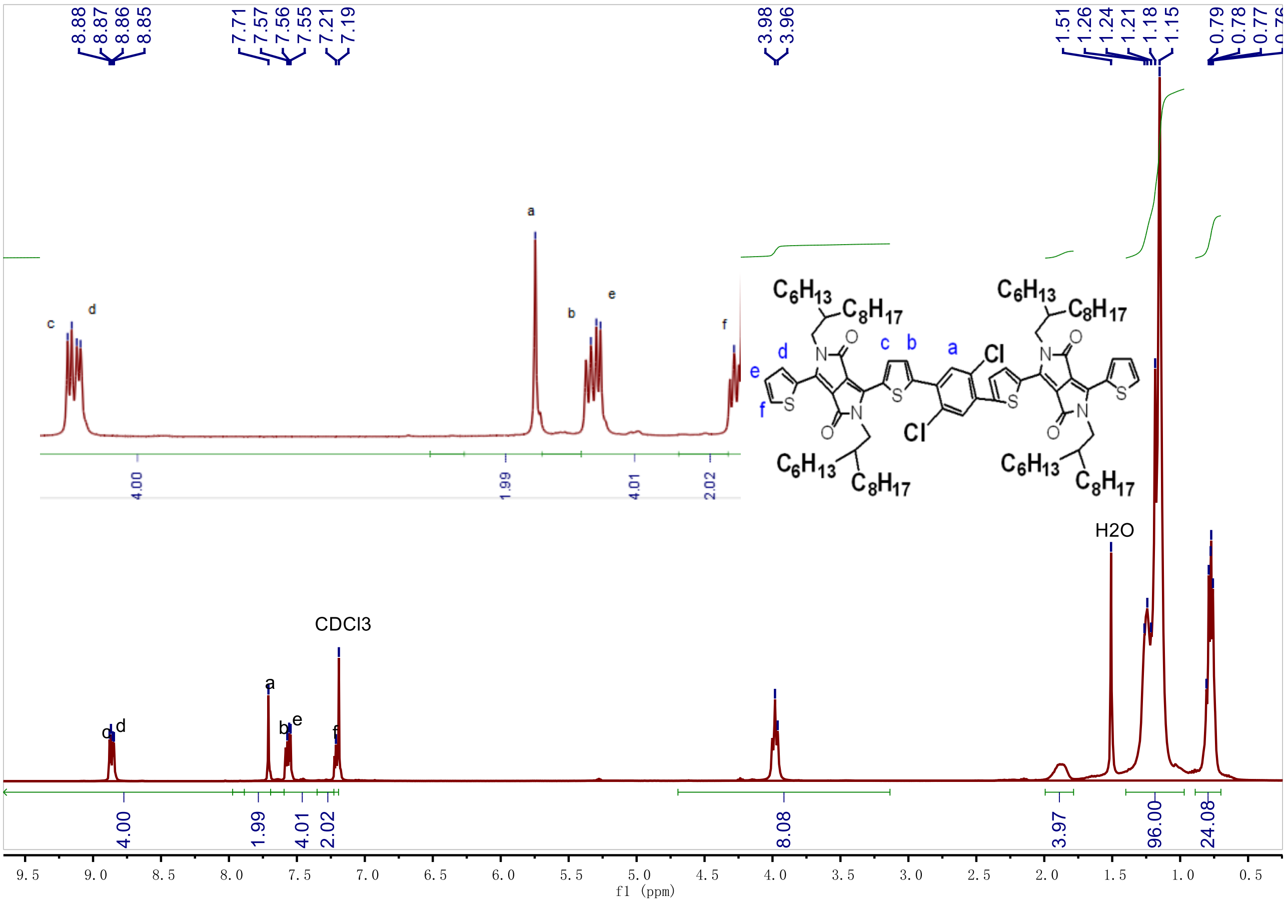


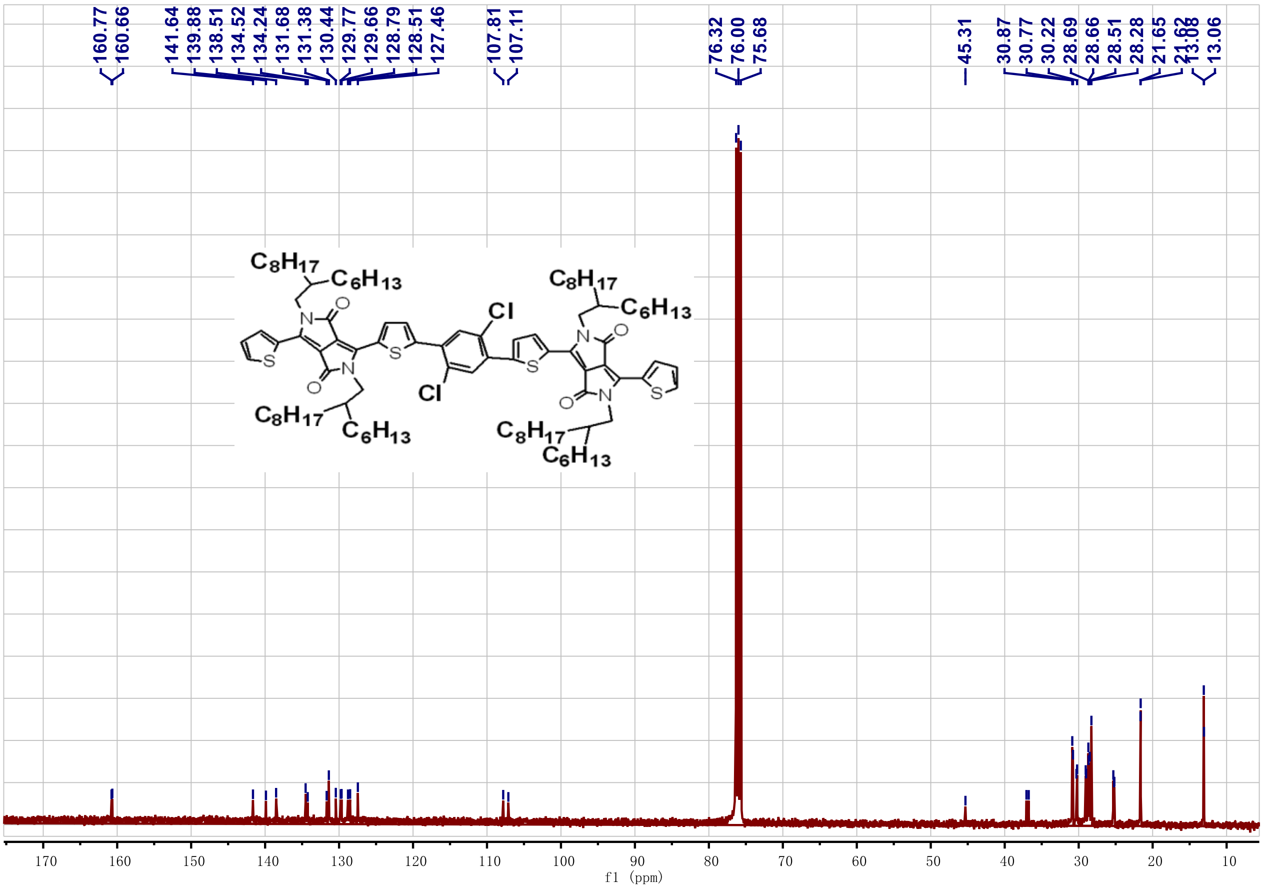


**Figure S9** $^{1}$H and $^{13}$C NMR spectra of **M8** in $CDCl_3$.

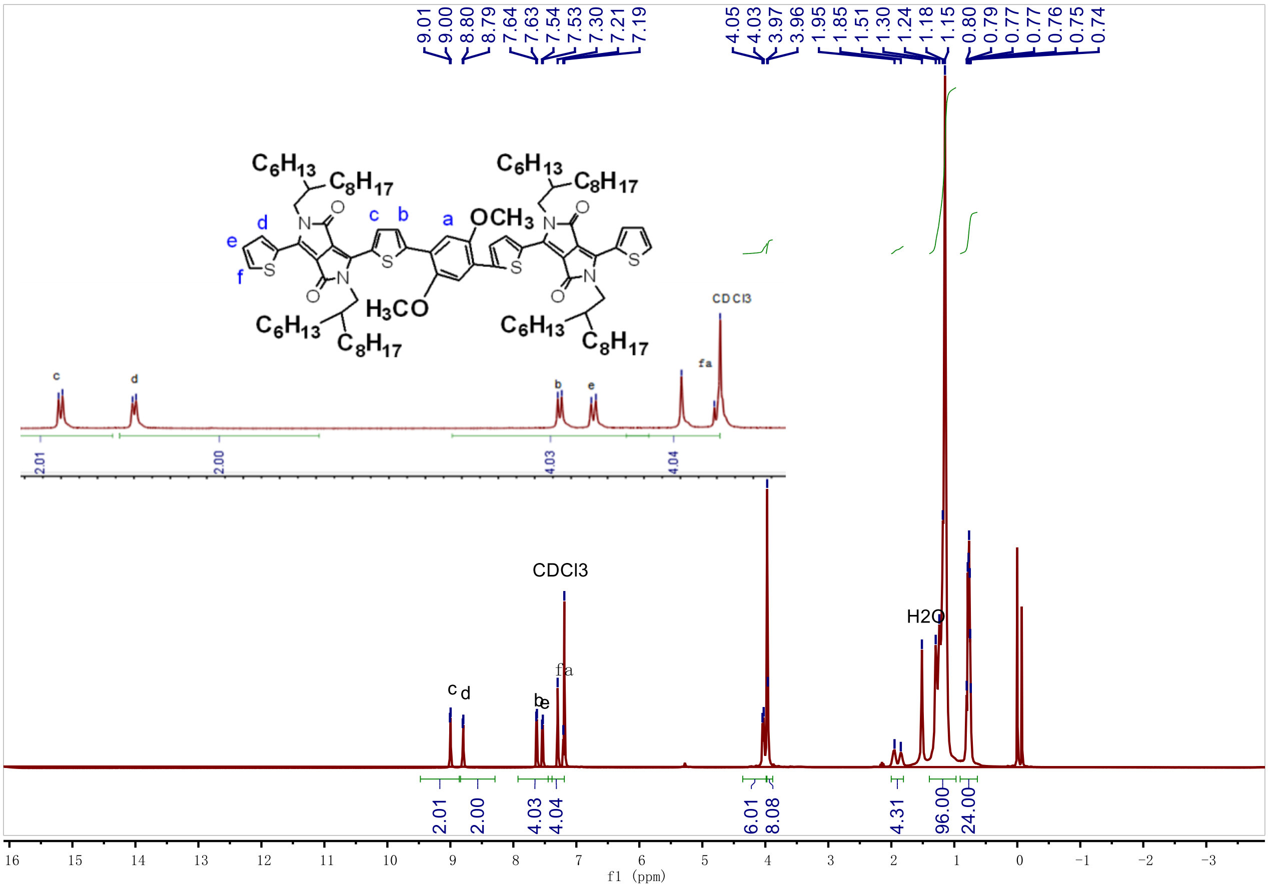


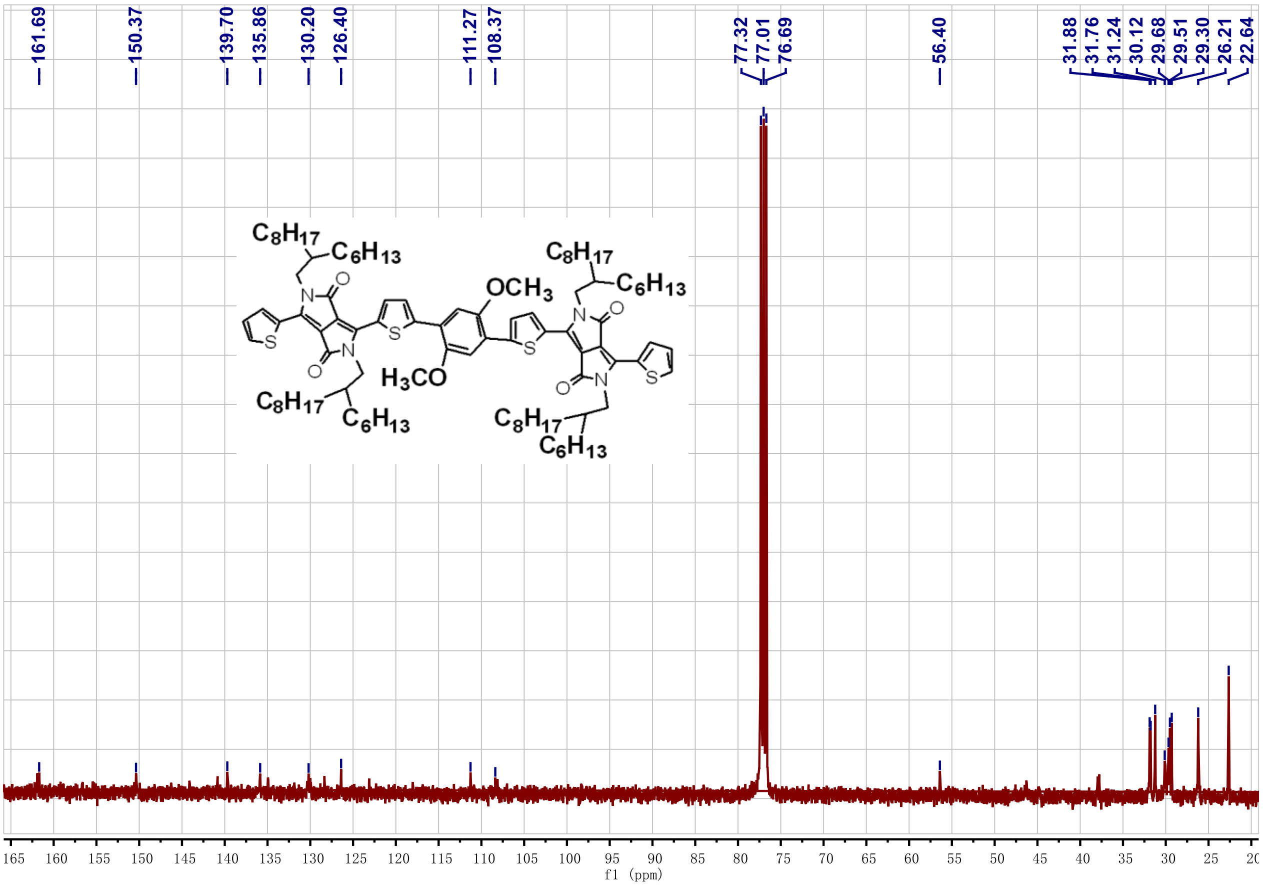


**Figure S10** $^{1}H$ and $^{13}C$ NMR spectra of **M9** in $CDCl_3$.

# 3.Maldi-TOF MS spectra

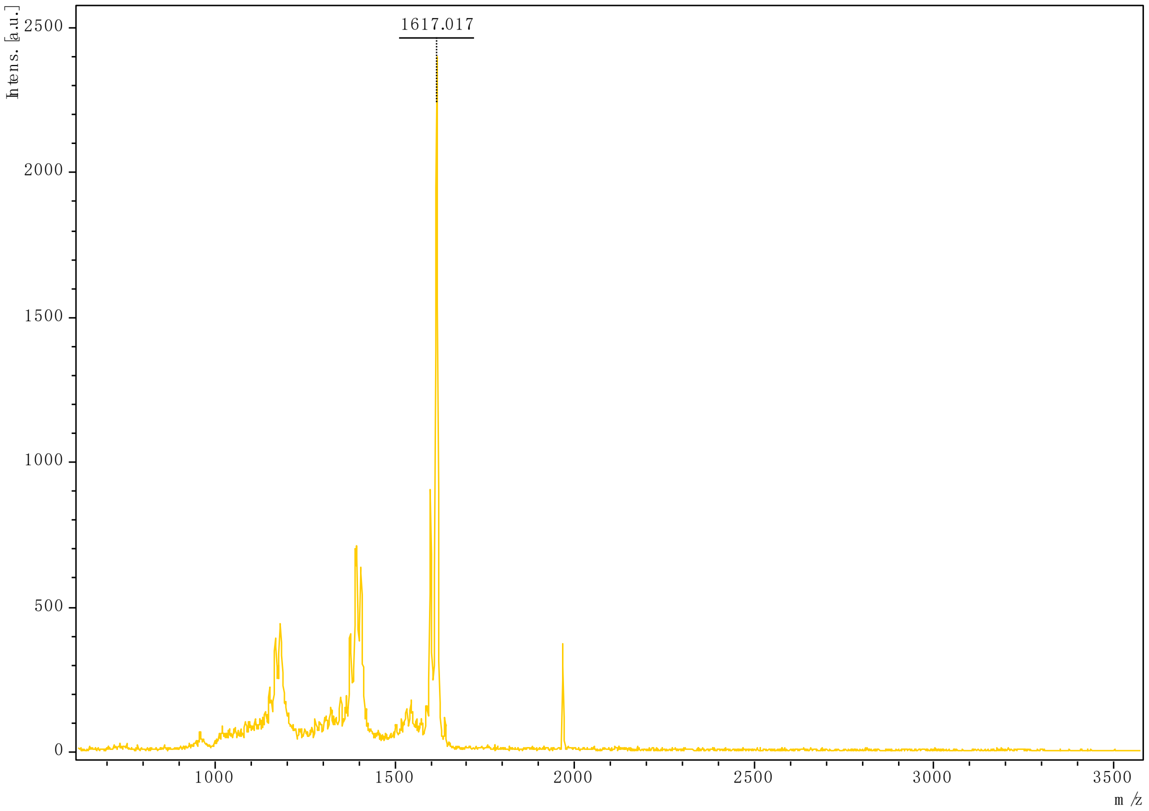


**FigureS11** MALDI-TOF MS spectrum of **M1**, calcd. 1617.5070, found 1617.0710 .

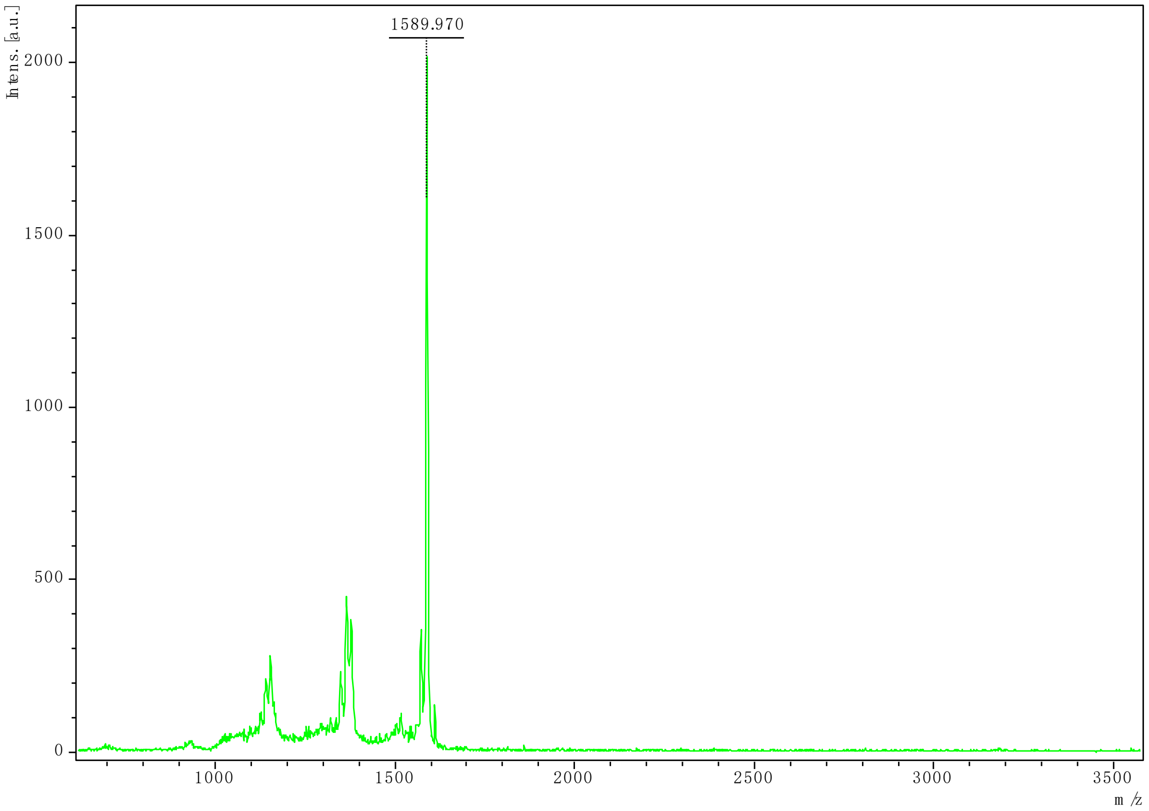


**FigureS12** MALDI-TOF MS spectrum of **M2**, calcd.1590.5004, found 1589.9700.

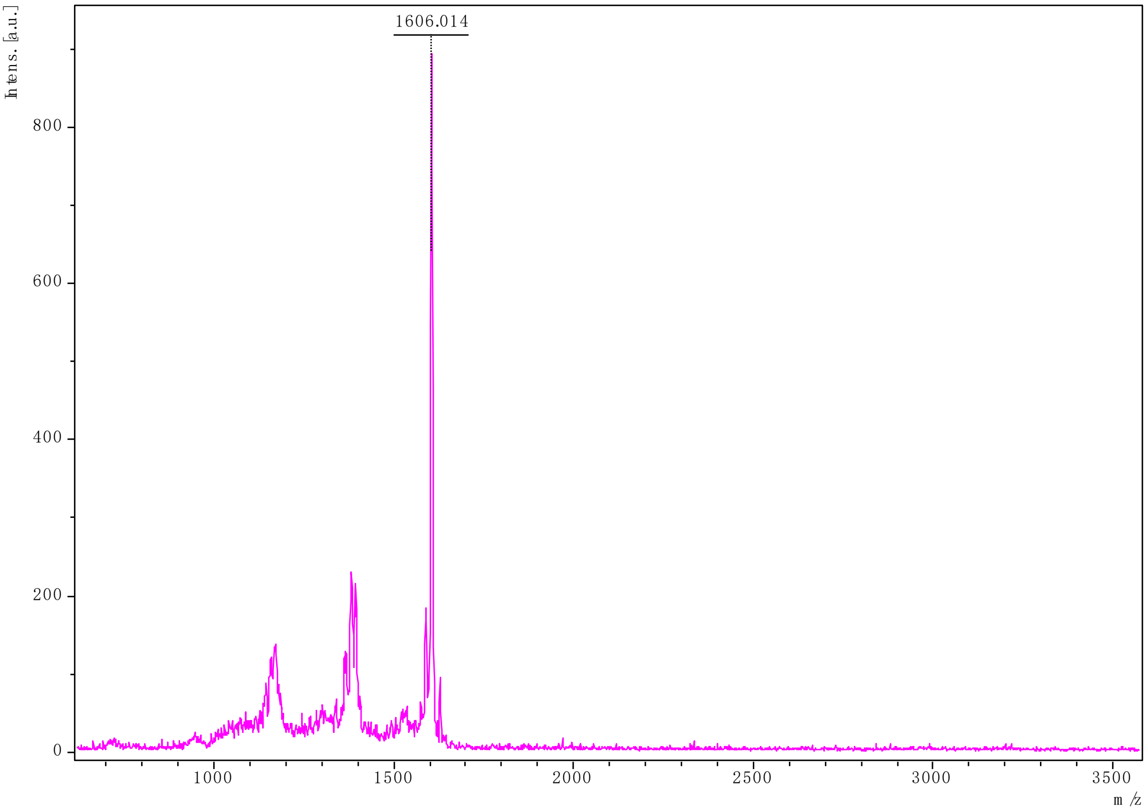


**FigureS13** MALDI-TOF MS spectrum of **M3**, calcd.1606.9520, found 1606.0140.

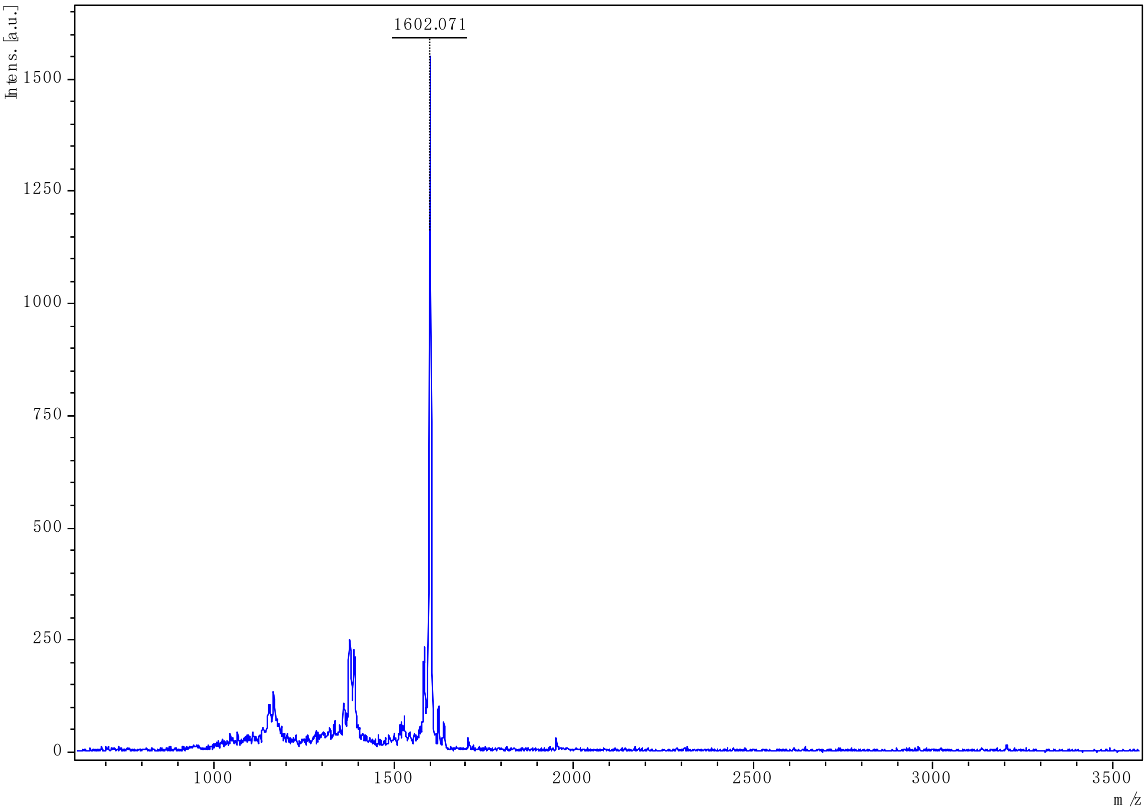


**FigureS14** MALDI-TOF MS spectrum of **M4**, calcd. 1602.5360, found 1602.0710.

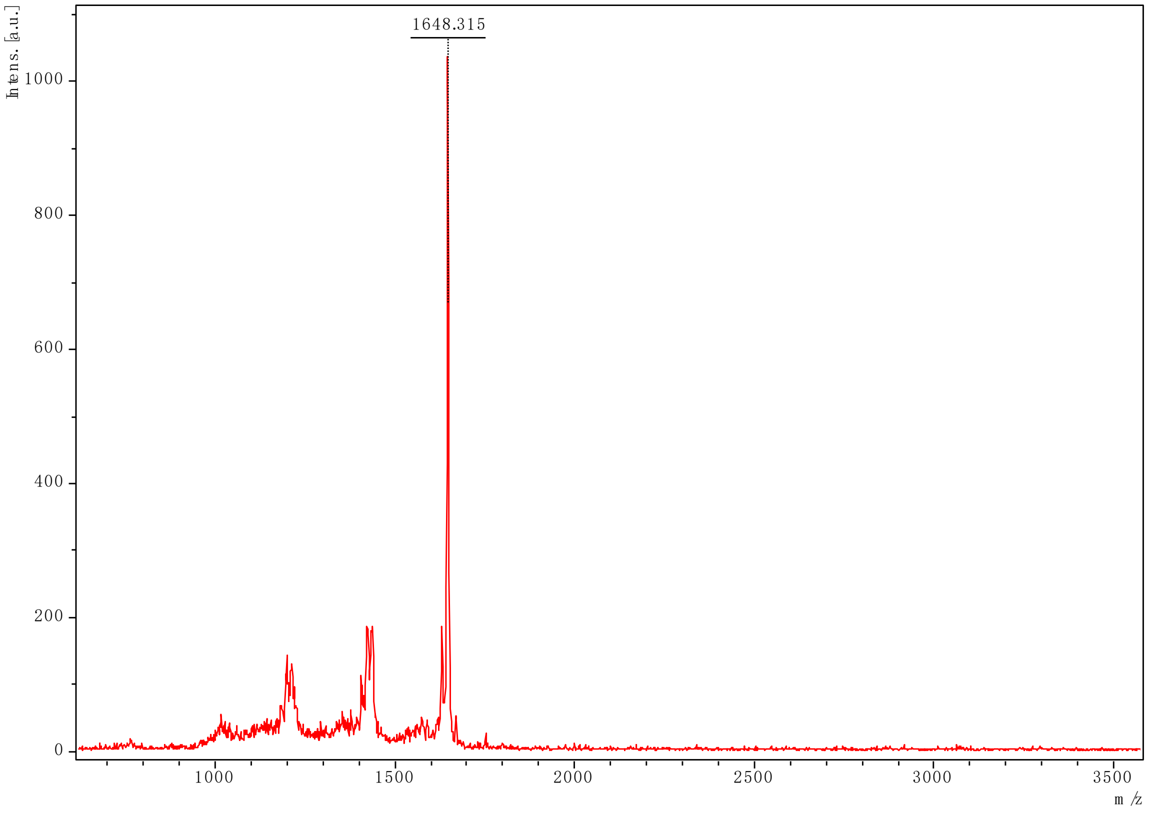


**FigureS15** MALDI-TOF MS spectrum of **M5**, calcd. 1648.6080, found 1648.3150.

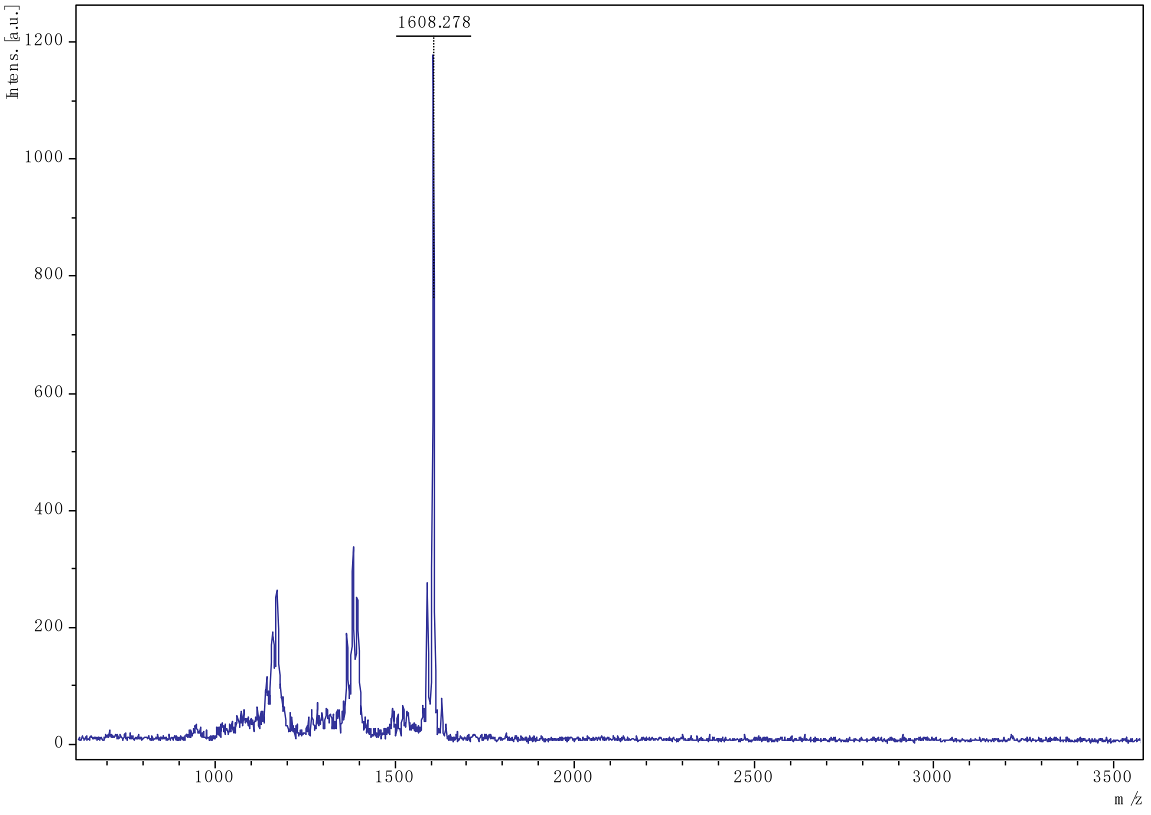


**FigureS16** MALDI-TOF MS spectrum of **M6**, calcd.1608.4900, found 1608.2780.

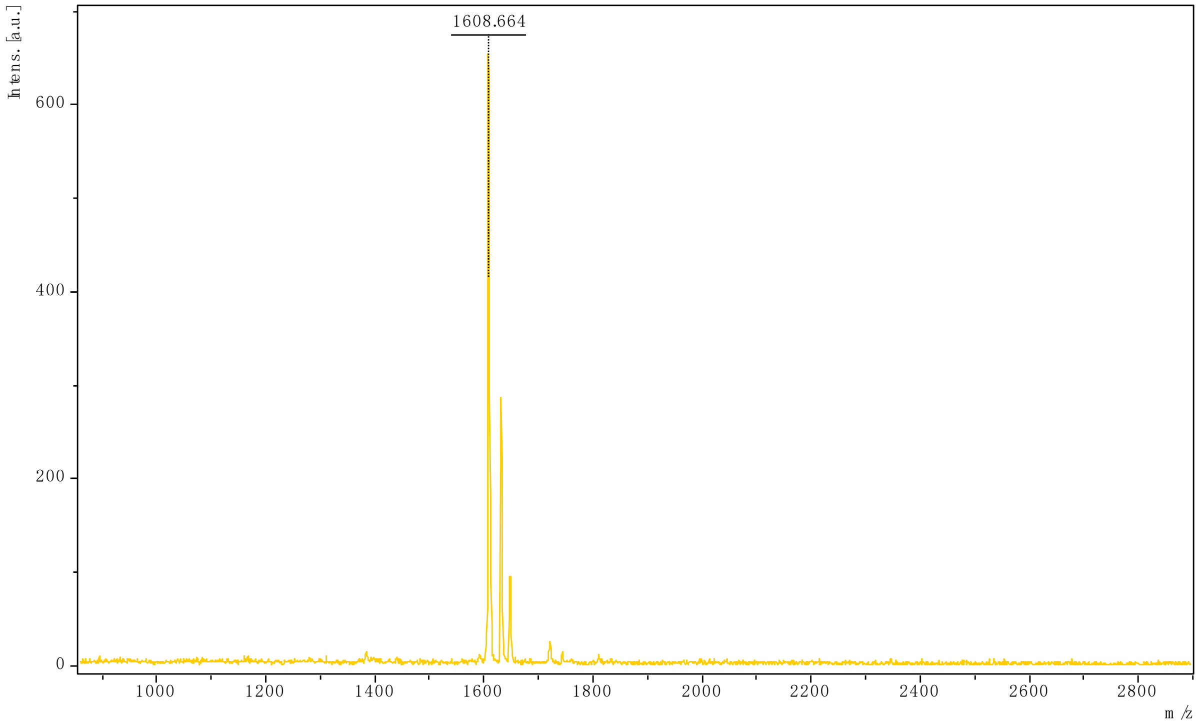


**FigureS17** MALDI-TOF MS spectrum of **M7**, calcd. 1608.4700, found 1608.6640.

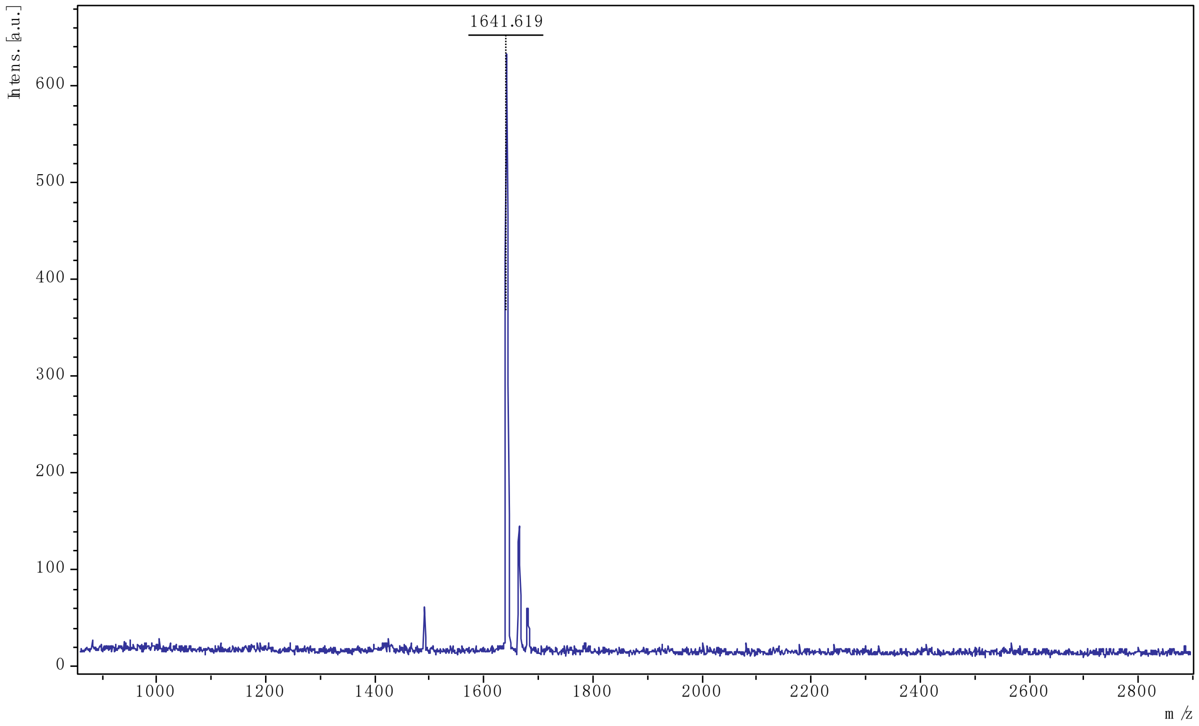


**FigureS18** MALDI-TOF MS spectrum of **M8**, calcd.1641.3800 , found 1641.6190 .

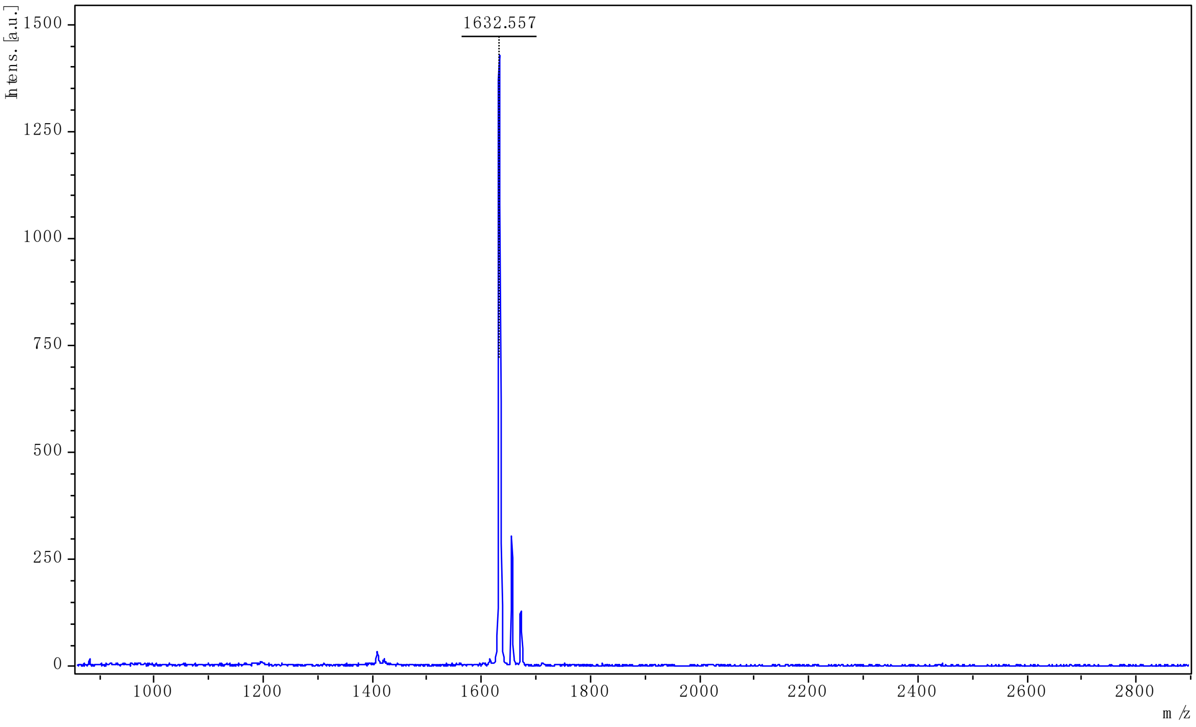


**FigureS19** MALDI-TOF MS spectrum of **M9**, calcd. 1632.5400, found 1632.5570.
